# Gadolinium-Based Magnetic Resonance Theranostic Agent with Gallic Acid as an Anti-Neuroinflammatory and Antioxidant Agent

**DOI:** 10.3390/antiox13020204

**Published:** 2024-02-05

**Authors:** Bokyung Sung, Dongwook Hwang, Ahrum Baek, Byeongwoo Yang, Sangyun Lee, Jangwoo Park, Eunji Kim, Minsup Kim, Eunshil Lee, Yongmin Chang

**Affiliations:** 1 ICT Convergence Research Center, Kyungpook National University, 80 Daehak-ro, Buk-gu, Daegu 41566, Republic of Korea; bksung@knu.ac.kr; 2 Department of Biomedical Science, School of Medicine, Kyungpook National University, 680 Gukchaebosang-ro, Jung-gu, Daegu 41944, Republic of Korea; dhwang@theranocure.com; 3 Theranocure Co., Ltd., 90 Chilgokjungang-daero 136-gil, Buk-gu, Daegu 41405, Republic of Korea; byang@theranocure.com (B.Y.); yunlee1903@knu.ac.com (S.L.); 4 Institute of Biomedical Engineering Research, Kyungpook National University, 680 Gukchaebosang-ro, Jung-gu, Daegu 41944, Republic of Korea; baxun@naver.com (A.B.); leees82@naver.com (E.L.); 5 Department of Medical & Biological Engineering, Kyungpook National University, 80 Daehak-ro, Buk-gu, Daegu 41566, Republic of Korea; 6 Korea Radioisotope Center for Pharmaceuticals, Korea Institute of Radiological & Medical Sciences, Seoul 01812, Republic of Korea; jangwoo@kirams.re.kr (J.P.); ejkim@kistep.re.kr (E.K.); 7 Center for Data Analytics Innovation, Office of National R&D Evaluation and Analysis, Korea Institute of S&T Evaluation and Planning, 1339, Wonjung-ro, Maengdong-myeon, Eumseong-gun 27740, Republic of Korea; 8 TARS Scientific, Nowon-gu, Seoul 01662, Republic of Korea; minsupkim.bio@gmail.com; 9 Department of Molecular Medicine, School of Medicine, Kyungpook National University, 680 Guchaebosang-ro, Jung-gu, Daegu 41944, Republic of Korea; 10 Department of Radiology, Kyungpook National University Hospital, 130 Dongdeok-ro, Jung-gu, Daegu 41944, Republic of Korea

**Keywords:** MRI, contrast agent, neuroinflammation, therapeutic, gallic acid, LPS, MD2, microglia

## Abstract

Studies in the field have actively pursued the incorporation of diverse biological functionalities into gadolinium-based contrast agents, aiming at the amalgamation of MRI imaging and therapeutic capabilities. In this research, we present the development of Gd-Ga, an anti-neuroinflammatory MR contrast agent strategically designed to target inflammatory mediators for comprehensive imaging diagnosis and targeted lesion treatment. Gd-Ga is a gadolinium complex composed of 1,4,7-tris(carboxymethylaza)cyclododecane-10-azaacetylamide (DO3A) conjugated with gallic acid (3,4,5-trihydroxybenzoic acid). Upon intravenous administration in LPS-induced mouse models, Gd-Ga demonstrated a remarkable three-fold increase in signal-to-noise (SNR) variation compared to Gd-DOTA, particularly evident in both the cortex and hippocampus 30 min post-MR monitoring. In-depth investigations, both in vitro and in vivo, into the anti-neuroinflammatory properties of Gd-Ga revealed significantly reduced protein expression levels of pro-inflammatory mediators compared to the LPS group. The alignment between in silico predictions and phantom studies indicates that Gd-Ga acts as an anti-neuroinflammatory agent by directly binding to MD2. Additionally, the robust antioxidant activity of Gd-Ga was confirmed by its effective scavenging of NO and ROS. Our collective findings emphasize the immense potential of this theranostic complex, where a polyphenol serves as an anti-inflammatory drug, presenting an exceptionally efficient platform for the diagnosis and treatment of neuroinflammation.

## 1. Introduction

A substantial body of experimental evidence underscores the critical role of neuroinflammation in initiating and perpetuating neurodegenerative processes across a spectrum of neurological diseases [1,2,3]. Activated glial cells serve as a hallmark of neuroinflammation in neurodegenerative diseases such as Alzheimer’s disease and Parkinson’s disease [4,5]. The brain houses representative glial cells, namely microglia and astrocytes. Microglia, present abundantly in the brain, possess a substantial quantity of toll-like receptor 4 (TLR4), an innate immune system receptor, on their cell membrane, while astrocytes exhibit comparatively lower levels [6,7,8,9]. TLR4 can initiate intricate inflammatory cascades through the recognition of pathogen-associated molecular patterns (PAMPs) or damage-associated molecular patterns (DAMPs) [10,11]. Lipopolysaccharide (LPS), a PAMP found in the outer membrane of Gram-negative bacteria, notably triggers TLR4 activation, making it a common stimulus to model neuroinflammation associated with neurodegeneration.

The TLR4 cascade induced by LPS translocates the nuclear factor kappa-light-chain-enhancer of activated B cells (NF-κB) into the nucleus, subsequently forming the nucleotide binding and oligomerization domain-like receptor family pyrin domain-containing protein 3 (NLRP3) inflammasome, generating cleaved caspase-1, and ultimately upregulating and releasing pro-inflammatory kinases, such as interleukin-1β (IL-1β) and inducible nitric oxide synthase (iNOS)—enzymes responsible for nitric oxide (NO) production [12,13]. Remarkably, during this process, LPS stimulation significantly induces oxidative stress, intensifying the inflammatory response [14,15,16]. Myeloid differentiation protein 2 (MD2) acts as a co-receptor for TLR4, playing a pivotal role in recognizing LPS and initiating a cascade of TLR4-dependent inflammatory responses in neuroinflammation. Inactivating TLR4 by binding the Gd complex to MD2 presents a potential avenue for effectively suppressing neuroinflammation stemming from the TLR4/NLRP3 inflammasome pathway.

Gallic acid (3,4,5-trihydroxybenzoic acid), an active polyphenol, presents compelling evidence supporting its efficacy as both an antioxidant and an anti-inflammatory agent [17]. Prevalent in various plant sources, this compound has undergone extensive research due to its potential health benefits. Its antioxidative properties are noteworthy for their capacity to neutralize detrimental free radicals within the body [18]. The antiradical activity of gallic acid primarily manifests through a single electron transfer (SET) mechanism [19], involving the transfer of electrons from the hydroxyl group to free radicals such as reactive oxygen species (ROS) and NO [20,21,22]. Several publications have reported that gallic acid derivatives can target MD2 and exhibit a high binding affinity [23,24]. Numerous studies have reported the regulatory role of gallic acid in the NLRP3 inflammasome pathway, demonstrating therapeutic potential in disease models such as inflammatory bowel disease and gouty arthritis. However, the focus has predominantly been on evaluating the therapeutic efficacy of natural products, and little attention has been given to exploring their functional application to diagnostic agents.

In this study, we aimed to design, synthesize, and evaluate an MRI theranostic agent with anti-neuroinflammatory properties, utilizing gallic acid. These pioneering agents integrate a molecular imaging component with a polyphenol serving as an anti-inflammatory drug, forming a unified combination platform. Employing this platform enables MR imaging to detect lesions at the molecular level in animal models induced with lipopolysaccharide (LPS)-stimulated inflammation. The Gd complex is intricately involved in the molecular mechanisms of the TLR4/NLRP3 inflammasome signaling pathways, resulting in a reduction of IL-1β and the inhibition of microglia activation. We hypothesize that this effect stems from Gd-Ga binding to MD2, thereby attenuating the TLR4/NLRP3 inflammasome pathway. Additionally, Gd-Ga, rich in electron donors, exhibits a profound inhibitory effect on free radicals such as ROS and NO, thereby mitigating NLRP3 inflammasome activation. In this study, we propose Gd-Ga as an MR theranostic agent with therapeutic potential against neuroinflammation, achieved through the direct scavenging of oxidative stress and the inhibition of TLR4 activation by binding to MD2, ultimately leading to a reduction in pro-inflammatory factors. This research not only sheds light on the intricate interplay of neuroinflammation but also presents a promising avenue for theranostic intervention.

## 2. Materials and Methods

### 2.1. Materials

Gallic acid, *N*-(3-Dimethylaminopropyl)-*N*′-ethylcarbodiimide hydrochloride (EDC∙HCl), 1-Hydroxybenzotriazole hydrate (HOBt Hydrate), *N*,*N*-Diisopropylethylamine (DIPEA), trifluoroacetic acid (TFA), and gadolinium (III) chloride hexahydrate (GdCl_3_∙6H_2_O) were purchased from Sigma Aldrich (St. Louis, MO, USA) and used without further purification. Other reagents and solvents were purchased from Tokyo Chemical Industry Co., Ltd. (Tokyo, Japan) and Ducksan Pure Chemicals Co., Ltd. (Ansan-si, Republic of Korea). Tri-tert-butyl 2,2′,2″-(10-(2-((2-aminoethyl)amino)-2-oxoethyl)-1,4,7,10-tetraazacyclododecane-1,4,7-triyl)triacetate (DO3A-^t^Bu-NH_2_) was prepared according to the methods described in the literature.

The lipopolysaccharides from Escherichia coli (LPS) purchased from Sigma Aldrich were used in in vivo and in vitro studies. Cell Counting Kit-8 (CCK-8) was purchased from Dojindo Laboratories (Kumamoto, Japan). BV-2 protein was purchased from R&D Systems, Inc. (Minneapolis, MN, USA). Bicinchoninic acid (BCA) Protein Assay Kit was obtained from Thermo Fisher Scientific (Waltham, MA, USA). Griess Reagent Kit was purchased from Invitrogen (Carlsbad, CA, USA). Radioimmunoprecipitation assay lysis buffer and immobilon-P membrane were purchased from Merck Millipore (Darmstadt, Germany). Protease and phosphatase inhibitor cocktail was purchased from Roche Diagnostics (Basel, Switzerland). Penicillin–streptomycin solution (PS) was purchased from Gibco (Carlsbad, CA, USA). Fetal bovine serum (FBS) was purchased from Hyclone (Waltham, MA, USA). Dulbecco’s Phosphate-Buffered Saline (DPBS) and Dulbecco’s modified Eagle’s medium (DMEM) were purchased from WELGENE (Daegu, Republic of Korea). All antibodies were purchased from Santa Cruz Biotechnology Inc. (Santa Cruz, CA, USA), Cell Signaling Technology (Beverly, MA, USA), and Abcam (Cambridge, UK). Pico EPD Western Reagent was purchased from ELPIS (Daejeon, Republic of Korea) and SuperSignal West Femto Maximum Sensitivity Substrate was obtained from Thermo Scientific (Waltham, MA, USA).

### 2.2. Instrument

The ^1^H NMR and ^13^C NMR (500 MHz) spectra were measured on an Avance III 500 (Bruker, Rheinstetten, Germany) at the Center for Instrumental Analysis, Kyungpook National University (KNU). A prep-HPLC system was run on an LC-forte/R (YMC, Kyoto, Japan) for the purification of compounds. High-resolution FAB-MS spectra were conducted at the Korea Basic Science Institute (KBSI) using a JMS-700 (Jeol, Tokyo, Japan) to confirm the identity of the compounds. ICP-MS (PerkinElmer Inc., Norwalk, CT, USA) was used for a quantitative analysis of the Gd complex. The phantom and in vivo studies were carried out by Signa Architect 3.0 T System (127.8 MHz, GE Healthcare, Milwaukee, WI, USA) and 9.4 T BioSpec 94/20 (400 MHz, Bruker, Germany). Absorbance was measured using SpectraMax^®^ i3 (Molecular Devices, San Jose, CA, USA) and Lambda 950 (PerkinElmer Inc., Waltham, MA, USA). Fluorescence data were obtained with the F-7000 (Hitachi, Tokyo, Japan). Chemiluminescence western imaging system was run on Amersham™ ImageQuant™ 800 (Cytiva, Amersham, UK).

### 2.3. Synthesis and Characterization

tri-tert-butyl 2,2′,2″-(10-(2-oxo-2-((2-(3,4,5-trihydroxybenzamido)ethyl)amino)ethyl)-1,4,7,10-tetraazacyclododecane-1,4,7-triyl)triacetate (1).

Gallic acid (11.61 mmol, 1.4 equiv) dissolved in DMF at 0 °C was added dropwise to the solution of EDC∙HCl (12.77 mmol, 1.6 equiv) and HOBt hydrate (12.77 mmol, 1.6 equiv) in DMF and stirred for 30 min. The reaction mixture was added to the solution of DO3A-^t^Bu-NH_2_ (8.13 mmol, 1 equiv) and dissolved in DMF. Afterward, DIPEA (23.22 mmol, 2.9 equiv) was added, and the mixture was stirred at r.t. overnight. The resulting mixture was evaporated under reduced pressure and washed with DCM and brine. The DCM layer was dehydrated using Na_2_SO_4_ and the drying agent was filtered. Open-column chromatography was performed under the condition of DCM/MeOH (96:4). The solvent was then removed and used in the next step without further purification.

2,2′,2″-(10-(2-oxo-2-((2-(3,4,5-trihydroxybenzamido)ethyl)amino)ethyl)-1,4,7,10-tetraazacyclododecane-1,4,7-triyl)triacetic acid (2).

**1** An amount of 4 g in an ice bath was added to the TFA/DCM (1:1, 80 mL) solution and further stirred at r.t. overnight. After the reaction, the solvent was removed under reduced pressure. The residue was dissolved in MeOH, and the resulting solution was added dropwise to diethyl ether. The precipitated solid was purified using flash chromatography (column: SNAP C18 30 g; flow rate: 25 mL/min; eluent A: water with 0.1% TFA; eluent B: MeCN with 0.1% TFA), resulting in the brown solid, **2** (3.51 mmol, **1** to **3**; 43%). The gradient condition was as follows: isocratic elution with 0% B for 2 min; 0 to 10% B for 12 min; 10 to 90% B for 2 min; isocratic elution with 90% B for 5 min. ^1^H NMR (500 MHz, CD_3_OD) δ 6.70 (d, *J* = 7.7 Hz, 2H), 3.91–3.46 (m, 8H), 3.31–3.28 (m, 2H), 3.24–2.78 (m, 18H). ^13^C NMR (126 MHz, CD_3_OD) δ 169.29, 169.26, 161.48, 161.20, 160.92, 160.64, 145.37, 136.80, 124.60, 106.60, 54.34, 53.20, 39.15, 38.75. HR-MS *m*/*z*: [M + H]^+^ Calcd for C_25_H_39_N_6_O_11_ 599.2677; found 599.2675.

Gd-DO3A-Ga (3).

**2** An amount of 1.67 mmol, 1 equiv, was dissolved in deionized water, and the pH was adjusted to 3 using a 1 M NaOH solution. GdCl_3_∙6H_2_O (1.17 mmol, 0.7 equiv) dissolved in deionized water was added to the solution of **2**, and 1 M NaOH solution was used to adjust the pH to 7. The reaction mixture was stirred at r.t. overnight. The product was evaporated under reduced pressure and purified using high-performance liquid chromatography (column: YMC-Actus Triart C18; flow rate: 12 mL/min) to yield **3** (0.38 g, 56%) as a brown solid. The gradient condition was as follows: isocratic elution with 0% B for 5 min; 0 to 15% B for 20 min; isocratic elution with 15% B for 10 min; 15 to 95% B for 3 min; isocratic elution with 95% B for 8 min. HR-MS *m*/*z*: [M + H]^+^ Calcd for C_25_H_36_GdN_6_O_11_ 754.1683; found 754.1687.

### 2.4. Relaxivity Measurement

T_1_ and T_2_ relaxation times of Gd complexes were measured using the IR-SE and CPMG pulse sequences on a 3.0 T MRI scanner. These relativity samples were prepared at concentrations of 1, 0.5, 0.25, 0.125, and 0.0625 mM. All experiments were performed with at least three identical samples (*n* = 3). For T_1_ measurements, 35 T_1_ images were acquired with different TIs ranging from 50 to 1750. T_2_ images were obtained at 16 different TEs from 7 to 111.7 ms. The image sequences were as follows for T_1_ measurements: IR; TR = 2000 ms; TE = 11 ms; TI = 50–1750 ms (35); matrix size = 320 × 192; NEX = 1; FOV = 40. T2 measurements: SE; TR = 1500 ms; TE = 7–111.7 ms (16); ETL = 1; matrix size = 256 × 192; number of averages = 1.

In the 9.4 T MRI study, T_1_ relaxation times were obtained using rapid acquisition with relaxation enhancement at variable TR (RARE-VTR) sequence. T_1_ images were acquired at various TR ranges (50, 75, 100, 150, 200, 300, 400, 500, 600, 800, 1000, 1500, 2000, 3000, 5000, 7500, and 10,000 ms). T_2_ relaxation times were determined using the multi-echo spin-echo (MESE) sequence with 70 different TEs (10–700 ms). The samples were prepared at the same concentration as in the 3.0 T MRI experiments. The image sequences were as follows for T_1_ measurements: RARE; TR = 50–10,000 ms (17); TE = 9 ms; Echo Train Length (ETL) = 2; matrix size = 256 × 256; number of averages = 1. T_2_ measurements: MESE; TR = 3000 ms; TE = 10–700 ms (70); ETL = 1; matrix size = 256 × 192; number of averages = 1.

T_1_ and T_2_ relaxation times were calculated using the non-linear least-square fit in MATLAB 2015a. The r_1_ and r_2_ relativities (s^−1^mM^−1^) are the R_1_ and R_2_ relaxation rate constants normalized to the concentration of the CAs and calculated by linear fitting of relaxation rates.

### 2.5. Kinetic Inertness and pH Stability Measurement

A total of 2.5 mM Gd complexes and 250 mM ZnCl_2_ solution dissolved in PBS were prepared. Subsequently, 2 μL of the ZnCl_2_ solution was added to 200 μL of Gd complex stock solution equivalently. The changes in T_2_ relaxation times were monitored for 72 h. R_2_(t) relaxation rates represent the inverse of T_2_ relaxation times. Values are expressed as mean ± SEM (*n* = 4). Gd-Ga was measured alongside commercial MR agents, GD-DOTA, Gd-DO3A-BT, Gd-DTPA, and Gd-DTPA-EOB, to compare their stability on 3.0 T MRI scanner. The MR sequences were as follows for T_2_ measurements: SE; TR = 2000 ms; TE = 11.7–89.5 ms (16); ETL = 1; matrix size = 256 × 256; number of averages = 1.

A total of 1 mM Gd-Ga was prepared in pH 1, 3, 5, 7, 9, and 11 buffer solutions. Each group consisted of at least three identical samples (*n* = 3). The pH stability experiment was conducted on a 3.0 MRI machine for 23 days, similar to the kinetic inertness study. To assess the stability of each sample based on pH, the changes in R_2_ values over time were compared. Values are expressed as mean ± SEM (*n* = 3). The T_2_ mapping parameters on MRI were as follows: SE; TR = 2000 ms; TE = 12.6–201.2 ms (16); ETL = 1; matrix size = 256 × 256; number of averages = 1.

### 2.6. DPPH and ABTS Assay [25,26]

A total of 1 mL of samples was prepared in different concentrations at 30, 20, 10, and 5 µM and dissolved in deionized water. A 200 µM DPPH solution was made by dissolving it in ethanol. Then, 500 µL of each sample was mixed with 500 μL of DPPH solution and incubated at r.t. for 30 min in the dark. To eliminate the inherent absorbance of the solvent, an extra 500 µL of each sample was diluted in 500 µL of ethanol. Samples that completed the reaction were placed in a 96-well plate in triplicate, each containing 200 µL. Absorbance values were measured at 517 nm using a microplate reader. Ascorbic acid was used as the control. The percentage of DPPH radical inhibition was according to the following Equation (1):

[(A_control_ − A_sample_)/A_control_] × 100 (1)

where A_control_ is the absorbance of the control, and A_sample_ is the absorbance of the sample treated with the drug. GraphPad Prism software (GraphPad Prism Software Inc., version 5.02, La Jolla, CA, USA) was employed for graphical analysis.

ABTS^+^∙ was generated by oxidating 20 mL of a 7 mM ABTS solution with 350 µL of a 140 mM potassium persulfate solution [26]. The mixture was allowed to react at r.t. for 16 h in the dark. The prepared 300 µL of ABTS^+^∙ solution was then diluted in 12 mL of ethanol. The assay involved mixing 10 µL of sample and 990 µL of ABTS^+^∙ solution (or ethanol). Absorbance values were measured at 734 nm after 35 min of mixing time. The percentage inhibition of absorbance was calculated and plotted as a function of the concentration of drugs using Equation (2) as follows:

(1 − A/A_0_) × 100 (2)

where A_0_ is the absorbance of the control and A is the absorbance of the sample treated with the drug. Analysis was conducted using the GraphPad Prism software.

### 2.7. Cell Culture and Cell Viability Measurement

BV-2 cells (microglial cells) were cultured following the manufacturer’s instructions. The cells were cultured in DMEM containing 10% FBS and 1% PS solution, maintaining a culture temperature of 37 °C in a humidified incubator with 5% CO_2_. To assess the cytotoxicity of Gd-Ga, BV-2 cells were plated in 96-well plates at 2 × 10^4^ cells/well. After 24 h of incubation, the medium was switched with a fresh serum-free medium containing various concentrations (0, 50, 100, 200, and 400 μM) of Gd-Ga, and cells were further incubated for 24 h. Subsequently, D-Plus™ CCK cell viability assay kit was added to each well, and cells were incubated for an additional 1 h. Absorbance was then measured at 450 nm using a microplate reader. The entire experiment was repeated 3 times, and each experiment was conducted once with *n* = 3.

### 2.8. Griess Assay and DCF-DA Assay

For verification of NO, production was measured in culture medium using the Gress reagent. Briefly, BV2 cells were seeded on a six-well plate at 5 × 10^5^ cells/well. After incubation for 24 h, the medium was replaced with serum-free medium containing LPS for 4 h, followed by the treatment of 400 μM Gd-Ga in the cells for 20 h. The cultured cells were harvested after washing with DPBS. Subsequently, the harvested cells were used to evaluate the expression of proteins through western blotting. The medium separated from the cultured cells was centrifuged at 4000× *g* for 2 min before using the supernatant for the Griess assay. Absorbance values at 560 nm were measured using a microplate reader (SpectraMax i3, Molecular Devices, San Jose, CA, USA).

In a separate study to establish the ROS scavenging effect, BV-2 cells (2 × 10^4^ cells/well) were plated into 96-well plates and incubated for 24 h. The medium was then changed to a serum-free medium with LPS and maintained for 4 h. Subsequently, Gd-Ga was treated in the cells for 20 h. Finally, after the H2DCFDA Kit (Thermo Scientific, Waltham, MA, USA, Thermo-D399) was applied and cultured for 45 min, the fluorescence values were measured at Ex/Em = 485/535 nm using a microplate reader.

### 2.9. Cytoplasmic and Nuclear Extracts for IκB and NF-κB

BV2 cells were seeded on a six-well plate at 5 × 10^5^ cells/well. Following incubation for 24 h, the medium was switched to a serum-free medium containing LPS for 4 h, and then 400 μM Gd-Ga was treated in the cells for 20 h. The cultured cells were harvested after washing with DPBS. Subsequently, nucleus and cytosol isolation were performed using NE-PER™ Nuclear and Cytoplasmic Extraction Reagents (Thermo Scientific, Waltham, MA, USA, #78835). Afterwards, the western blotting process was conducted.

### 2.10. Western Blot

Proteins were homogenized in an RIPA lysis buffer containing a protease and phosphatase inhibitor cocktail and centrifuged at 13,000× *g* for 15 min at 4 °C. Protein concentrations were determined using the Pierce BCA Protein Assay Kit. Proteins (15 μg in in vitro; 40 μg in in vivo) were loaded onto 8.5% SDS-PAGE and transferred onto a PVDF. The membranes were blocked in 3% BSA Tris-buffered saline (TBS) solution containing 0.05% Tween 20 (TBS-T) at r.t. for 2 h. Membranes were incubated with primary antibodies diluted in a 3% BSA buffer overnight at 4 °C.

The primary antibodies used in the experiment were NLRP3 (1:1000, ab270449, Abcam, Cambridge, UK), ASC (1:1000, #67824, Cell Signaling Technologies, Danvers, MA, USA), IL-1β (1:1000, #12242, Cell Signaling Technologies), Iba1 (1:1000, ab178846, Abcam), GFAP (1:2000, #3670, Cell Signaling technologies), iNOS (1:1000, #13120, Cell Signaling Technologies), β-actin (1:2000, sc47778, Santa Cruz Biotechnology, Dallas, TX, USA), NF-κB (1:1000, #6956, Cell Signaling Technologies), IκB-α (1:1000, #4814, Cell Signaling Technologies), pIκB-α (1:1000, #2859, Cell Signaling Technologies), Lamin B1 (1:1000, #12586, Cell Signaling Technologies), and α-Tubulin (1:1000, #2144, Cell Signaling Technologies).

Subsequently, the membranes were incubated with a secondary antibody (1:2000 dilution; Cell Signaling Technology) at r.t. for 1 h. Band signals for proteins were obtained in a chemiluminescence western imaging system using PicoEPD Western Reagent (ELPIS-Biotech, Daejeon, Korea) or SuperSignal West Femto Maximum Sensitivity Substrate (Thermo Fisher Scientific, Waltham, MA, USA). Western blot images were analyzed using ImageJ software (version 1.50i). Full bands for all western blots are represented in Figures S13–S15.

### 2.11. Biodistribution Method

Gd-Ga (0.1 mmol/kg) was administered intravenously as a bolus in male C57BL/6J mice (7–8 weeks old), and the mice were sacrificed at 10 min, 2 h, and 24 h after administration. The extracted organs were digested with a solution of 70% HN_3_ and 30% H_2_O_2_ over 100 °C for 2 h to obtain clear liquid samples, which were diluted in a 3% nitric acid solution. After filtering all sample organs (heart, lung, liver, gallbladder, spleen, kidney, and intestine), quantitative analysis of Gd ions was performed using an ICP spectrometer (PerkinElmer Inc., Norwalk, CT, USA). Biodistribution data were represented as %ID, defined as the percentage of the injected dose. Values are expressed as the mean ± SD, *n =* 3.

### 2.12. LPS-Induced Meuroinflammation Mouse Model

To establish the LPS-induced mouse model, male C57BL/6J mice (7–8 weeks old) were procured from Hana Biotech (Pyeongtaek, Republic of Korea). The mice were housed under automatically controlled conditions with a 12 h light/dark cycle at a temperature of 23 ± 1 °C and 50 ± 10% humidity. All animal experiments were approved and conducted according to the Institutional Animal Care and Use Committee (IACUC) of Kyungpook National University (protocol code: KNU-2022-0394). The mice were divided into the following treatment groups for the western blot study:

(I) i.c.v. saline group (control).
(II) i.c.v. LPS group (LPS.)
(III) i.c.v. LPS group treated with Gd-Ga (LPS + Gd-Ga).

The i.c.v. injections of LPS and the control group injected with saline were administered on one day using 71,000 Automated Stereotaxic Instrument (RWD Life Science Co., Ltd., Shenzhen, China) and stereotaxic coordinates (−2 mm dorsal/ventral, −1 mm lateral, and −0.2 mm anterior/posterior from the bregma). For groups (I) and (II), the same volume of saline was intravenously injected.

### 2.13. In Vivo MR Imaging Method

All MR images were acquired before and after the administration of Gd CAs (0.2 mmol/kg of body weight) in LPS-induced mice under isoflurane anesthesia (~3% in O_2_ gas) on a 9.4 T MRI scanner (BioSpec, Bruker, Billerica, MA, USA). The T_1_-weighted images were as follows: TR = 700 ms; TE = 7 ms; ETL = 1; matrix size: 128 × 128; number of averages = 4; slice thickness = 0.4 mm. The resulting T_1_-weighted MR images were used to evaluate the brain enhancement and analyzed using imageJ software. Signal intensity (SI) in the hippocampus and cortex regions of mice, drawn through regions of interest (ROI), was quantified. The noise in each image was assessed from the SI of the background. The signal-to-noise ratio (SNR) was defined as the ratio of SI to noise. The ∆SNR values of the hippocampus and cortex area were calculated with the following Equation (3):

∆SNR = SNR_post_ − SNR_pre_. (3)

### 2.14. Immunofluorescence Staining

After isolating the brains from LPS-induced mice, the whole brains were immersed in a 4% PFA solution and fixed for 1 day at 4 °C. Subsequently, the fixed brains were paraffinized and sliced into 5 μm thick sections for the immunofluorescence staining. Sections were deparaffinized at 65 °C for 1 h and rehydrated using xylene and various diluted ethanol solutions ranging from 100% to 30%. The sections in sodium citrate buffer were subjected to microwave heating for antigen retrieval. Blocking of sections was achieved using a 5% BSA buffer containing 5% NGS (pH 7.6). Incubation with an anti-Iba-1 (1:400, ab178846, Abcam) and GFAP (1:400, #3670, Cell Signaling Technologies), was carried out at 4 °C overnight. After being washed with a TBS solution, the sections were incubated with Alexa Fluor 555 Donkey Anti-mouse IgG (1:150, Cell signaling Technologies) for 1.5 h in the dark. Subsequently, the brain slices underwent washing with a TBS solution and were mounted using VECTASHIELD^®^ Antifade Mounting Medium with DAPI (H-1300, Vector Laboratories, Burlingame, CA, USA). The coverslips were applied, and the slices were dried in the dark. Fluorescence images were captured using a fluorescent microscope (ECLIPSE Ti, Nikon, Tokyo, Japan) and a confocal laser scanning microscope (LSM 800 with AiryScan, Carl Zeiss, Jena, Germany).

### 2.15. Docking Study

The crystal structure of the human MD2 and TRL4-MD2 complexes was obtained from the PDB database website (https://www.rcsb.org, with PDB IDs 2E56 and 3FXI, respectively, accessed on 20 November 2023). For molecular modeling simulations, the Open Babel program (http://openbabel.org/, accessed on 20 November 2023) was utilized to add missing hydrogen atoms to the crystal structure and assign optimal charges for the polar hydrogen atoms. All ligands containing Gd complexes were constructed into three-dimensional structures and assigned appropriate protonation states using the RDKit library (https://www.rdkit.org/, accessed on 20 November 2023). Protein–ligand docking simulations were conducted using the custom Autodock Vina application (https://vina.scripps.edu/, accessed on 20 November 2023), which includes newly developed molecular mechanics and artificial intelligence-based scoring functions. Molecular dynamics simulation was performed using GROMACS (https://www.gromacs.org, accessed on 20 November 2023). All molecular modeling images were created with the open-source Pymol, and the PLIP application (https://github.com/pharmai/plip, accessed on 20 November 2023) was employed to analyze protein–ligand interactions.

### 2.16. Binding Affitity for MD2 Protein and Gd Complex

For the binding assessment, a UV–vis spectrophotometer was employed. Solutions of 10 µM Gd-Ga and 500 µM MD2 were prepared in PBS buffer (pH 7.4). After adding 3 µL of the MD2 stock to 3 mL of the Gd-Ga solution, dilution was directly performed in optical cell. Protein was added steadily until the saturation point was reached. The resulting absorbance values were presented as spectra of wavelength and analyzed through nonlinear fitting of one site-specific binding using GraphPad Prism software.

### 2.17. Statistical Analysis

All experiments were performed at least in triplicate, and the data were presented as the mean ± standard error of the mean (SEM) or standard deviation (SD). Statistical comparisons between two groups were validated by Mann–Whitney U test or independent *t*-test, with statistical significance accepted at *p* < 0.05.

## 3. Results

### 3.1. Synthesis Ans Characterization

**Figure 1:**
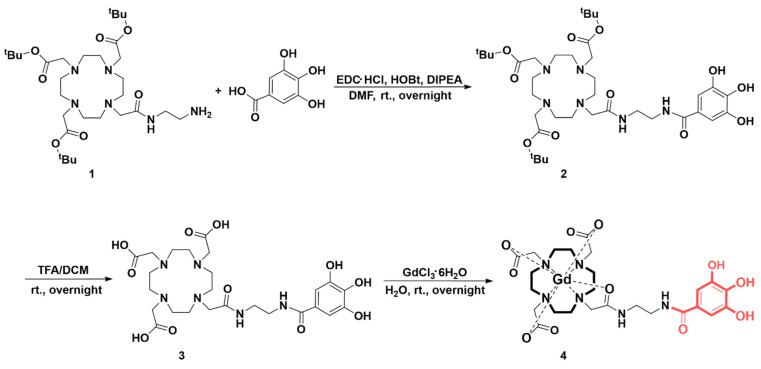
Synthesis of the Gd complex of the DO3A–gallic acid conjugate.

In Figure 1, the synthetic procedure for the Gd complex, abbreviated as Gd-Ga, is depicted. The synthesis of DO3A-^t^Bu-NH_2_ followed procedures featured in the literature [27]. Protected DO3A compounds, including DO3A-^t^Bu ester, were predominantly employed in the synthesis of DO3A-based agents, with the removal of protected groups in the subsequent step. Compound **1** was formed through the amice bonding of DO3A-^t^Bu-NH_2_ with gallic acid. While various reagent combinations are available for amide bind formation, EDC∙HCl and HOBt hydrate were chosen as the coupling agents in this study. **1** was roughly separated by an open column and used for ligand synthesis without further purification. For ligand preparation, **1** underwent deprotection with TFA diluted in DCM, a scavenger commonly used to remove tert-butyl ester. Compound **2** was separated using flash chromatography with a C18 reverse-phase column, resulting in a brown solid (43% yield). Subsequently, **2** was coordinated through a reaction with GdCl_3_ to produce compound **3**, Gd-Ga. This compound was synthesized and purified using high-performance liquid chromatography with a C18 reverse-phase column, affording it a brown solid (63% yield). The completion of all reactions was verified using thin-layer chromatography (TLC) and LC-MS (ESI positive mode). **2** was characterized by ^1^H NMR, ^13^C NMR, and HR-MS, while Gd-Ga was exceptionally analyzed using HR-MS and HPLC without NMR data (Figures S1–S4). The final compound’s purity was determined to be higher than 95% using HPLC (Figure S5). After purifying Gd-Ga, the presence of free gadolinium residues was confirmed through absorbance changes using arsenazo (III) solution (NaOAc buffer, pH 7.2, 0.01 M) (Figure S6). The absorption and emission spectra of Gd-Ga are shown in Figure S7, with the maximum λ_em_ observed at a wavelength of 353 nm at λ_ex_ 250 nm (Figure S7).

### 3.2. Physicochemical Characterization

The longitudinal (r_1_) and transverse (r_2_) relativities of Gd-Ga were acquired from clinical 3.0 T and preclinical 9.4 T MRI systems (Table 1). Several concentrations of phantoms were prepared with Gd-Ga dissolved in deionized water. The r_1_ and r_2_ relativities of Gd-Ga were 4.04 ± 0.17 and 4.82 ± 0.21 mM^−1^s^−1^, respectively, higher than those of Gd-DOTA, which measured 3.40 ± 0.07 and 3.88 ± 0.16 mM^−1^s^−1^ at 3.0 T MRI. In the preclinical 9.4 T MRI system, the relativities of Gd-Ga were 4.14 ± 0.11 and 4.78 ± 0.07 mM^−1^s^−1^, while those of Gd-DOTA were 3.63 ± 0.07 and 4.77 ± 0.11 mM^−1^s^−1^. A higher relativity of CAs would give equivalent signal enhancement at a lower dose compared to CAs with lower relativity values. This characteristic can address Gd-induced safety concerns and contribute to improving the efficacy of targeted CAs [28].

### 3.3. Kinetic Inertness and pH Stability

**Figure 2:**
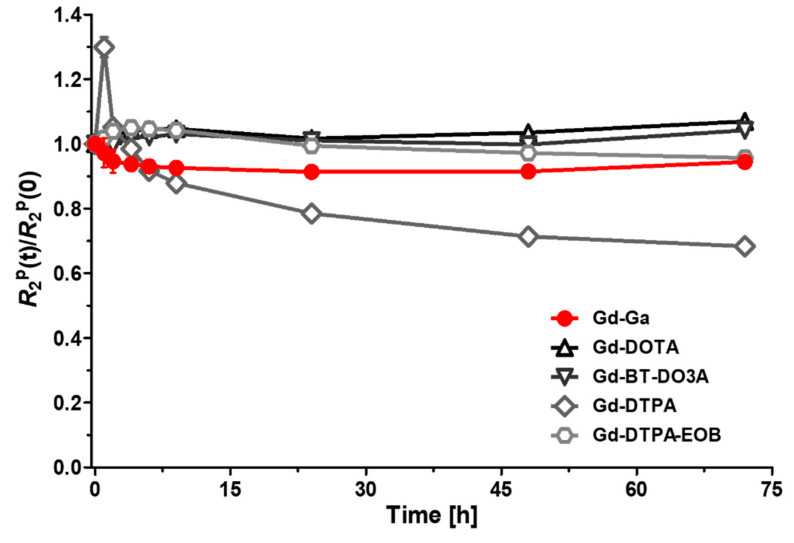
Evaluation of longitudinal relaxation rates R_1_^P^(t)/R_1_^P^(0) for Gd-Ga and various MR contrast agents (Gd-DOTA, Gd-BT-DO3A, Gd-DTPA, and Gd-DTPA-EOB) on a 3.0 T MR system up to 72 h. Data are presented as the mean ± SD, *n* = 4.

The kinetic inertness of Gd-Ga against commercial MR Cas (Gd-DOTA, Gd-BT-DO3A, Gd-DPTA, and Gd-DTPA-EOB) was verified, as shown in Figure 2. The relaxation rate measurement was taken over a period of 0–96 h in the presence of Zn^2+^ ion. The transmetallation of Gd^3+^ and Zn^2+^ was confirmed by measuring the change in R_2_^P^ values. Transmetallation was expressed as the normalized transverse relaxation rate $R_{2}^{P}$(t)/$R_{2}^{P}$(0) over time [29]. No significant changes in the R_2_ relaxation rate were observed for 3 days. However, acyclic Gd-DTPA persistently resulted in $R_{2}^{P}$(t)/$R_{2}^{P}$(0) < 1 over time, indicating transmetallation from paramagnetic Gd ion to diamagnetic Zn ion. It is well established that the MR Cas with macrocyclic chelates exhibit greater stability compared to those with a linear structure [30,31].

The pH stability of Gd-Ga was evaluated by measuring the R_2_ relaxation time over time at various pH conditions (Figure S8). The R_2_ values of Gd-Ga remained constant between 5 and 8 S^−1^ across a range of pH levels from 1 to 11 for 23 days. In contrast, the R_2_ relaxation time of Gd-DOTA changed gradually due to the dissociation of the Gd ion from the DO3A chelate under very strong conditions (Figure S8). While macrocyclic Gd chelates are generally kinetically inactive, the dissociation of Gd ions can occur if they are thermodynamically unstable under highly acidic conditions [32].

### 3.4. Free Radical Scavenging Activity

**Figure 3:**
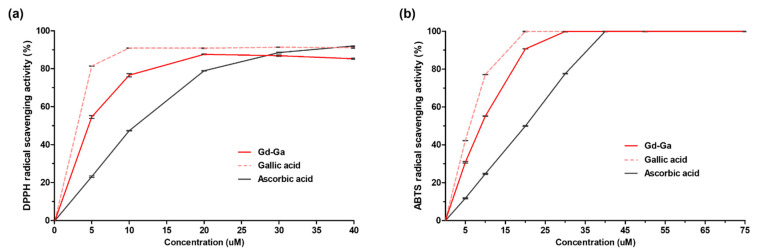
Evaluation of the antioxidant activities of Gd-Ga in comparison to gallic acid and ascorbic acid. (**a**) DPPH and (**b**) ABTS radical scavenging activities of Gd-Ga, gallic acid, and ascorbic acid. Data are presented as the mean ± SEM, *n* = 3.

The antioxidant efficacy of Gd-Ga was assessed using the DPPH and ABTS assay. Ascorbic acid, commonly known as vitamin C, served as the standard drug for antioxidant activity comparison. Gallic acid was included in the study to verify how well the synthesized Gd complex maintained the efficacy of the original polyphenols. The EC_50_ values of drugs were calculated and reported in Table 2. In Figure 3, the free radical scavenging effect followed the order of gallic acid > Gd-Ga > ascorbic acid in both the DPPH and ABTS assay. The EC_50_ value of Gd-Ga was 4.01 µM within a concentration range of 0–40 µM in the DPPH assay [33]. In the ABTS assay, the EC_50_ value of Gd-Ga was 8.03 µM within the range of 0–100 µM. The results of both experiments indicated that the higher radical scavenging activity of Gd-Ga compared to that of ascorbic acid. Although Gd-Ga exhibited lower activity than gallic acid, it was anticipated to possess sufficient antioxidant activity.

### 3.5. Intracellular Antioxidant Effect of Gd-Ga on LPS-Induced BV-2 Cells

Before assessing the intracellular antioxidant effect on LPS-induced BV-2 cells, cytotoxicity was evaluated based on cell viability relative to control cells. Cell viability above 80% is rated as non-cytotoxic, within 60–80% as weak cytotoxicity, 40–60% as moderate cytotoxicity, and below 40% as strong cytotoxicity in in vitro cytotoxicity assessment [34,35]. To investigate the impact of Gd-Ga on cytotoxicity, BV-2 cells were employed, and cell viability was assessed using a CCK-8 assay kit. In Figure S9, the cell viability of Gd-Ga in BV-2 cells showed a non-toxic tendency even up to a concentration of 400 μM.

**Figure 4:**
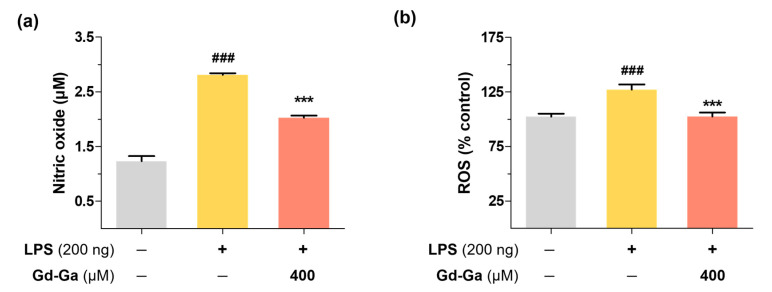
Effects of Gd-Ga on LPS-induced NO and ROS production in BV2 cells. BV-2 cells were treated with LPS (200 ng/mL) alone and with 400 μM Gd-Ga for 20 h. (**a**) The concentration of nitrite in the medium was measured using Griess assays. Data are presented as the mean ± SEM, *n* = 3. (**b**) Cellular ROS generation was measured by DCFDA assay. Data are presented as the mean ± SEM, *n* = 7. ^###^ *p* < 0.001, significant difference from the control, *** *p* < 0.001, significant difference from LPS.

Numerous studies provide evidence that oxidative stress is a key mediator of various neuronal disorders [36,37]. LPS can induce the excessive formation of ROS and RNS, leading to oxidative stress and subsequently accelerating neuroinflammation by affecting signaling pathways and damaging cells and tissues [33,38]. As shown in Figure 4, the NO scavenging effect of 400 μM Gd-Ga was evaluated using the Griess assay in LPS-induced BV-2 cells, resulting in reduced NO levels. Additionally, Figure 4 demonstrates that the Gd complex decreased ROS levels in the DCF-DA assay used to measure intracellular ROS formation. Gd-Ga exhibited an antioxidant effect, reducing both NO and ROS in LPS-induced BV-2 cells.

### 3.6. Effects of Gd-Ga on IκB Phosphorylation and NF-κB Translocation in LPS-Induced BV-2 Cells

**Figure 5:**
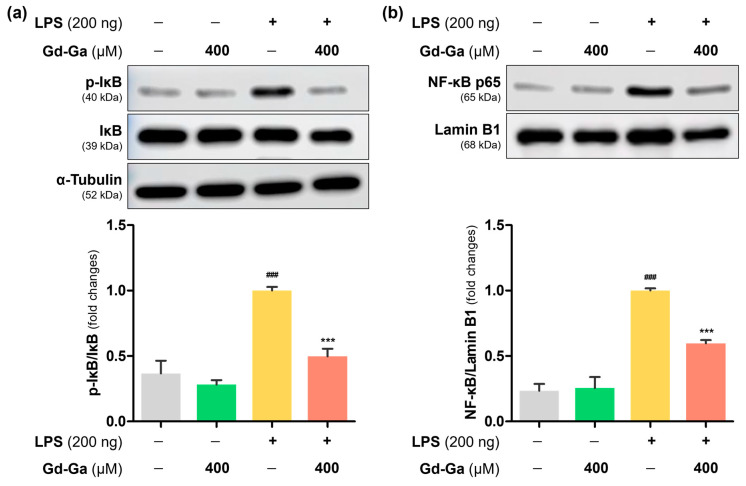
Effects of Gd-Ga on p-IκB, IκB, and nuclear NF-κB p65 protein levels in LPS-stimulated BV-2 cells. (**a**) The ratio of p-IκB to IκB and representative images of p-IκB and IκB. (**b**) Translocation of NF-κB confirmed through nuclear extraction. Bars represent relative protein quantification of p-IκB/IκB normalized to α-tubulin, and NF-κB normalized to Lamin B1, respectively. Data are shown as the mean ± SEM, *n* = 4. ^###^ *p* < 0.001 compared to the control group; *** *p* < 0.001 compared to the LPS-induced group.

NF-κB, a crucial transcription factor that generates pro-inflammatory molecules upon nuclear translocation, is regulated by IκB in normal conditions, preventing its entry into the nucleus. However, LPS stimulation promotes the phosphorylation of IκB, releasing NF-κB. Gd-Ga treatment resulted in significantly lower p-IκB expression compared to the levels induced by LPS in cytosol of BV-2 cells (Figure 5a). The LPS-treated group exhibited an increasing trend in the nuclear translocation of NF-κB. However, in the group treated with Gd-Ga, NF-κB levels in the nucleus significantly decreased (Figure 5b). Based on these results, this indicated that Gd-Ga inhibited the nuclear translocation of NF-κB by suppressing the phosphorylation of IκB.

### 3.7. Anti-Inflammatory Effect of Gd-Ga in LPS-Induced BV-2 Cells

**Figure 6:**
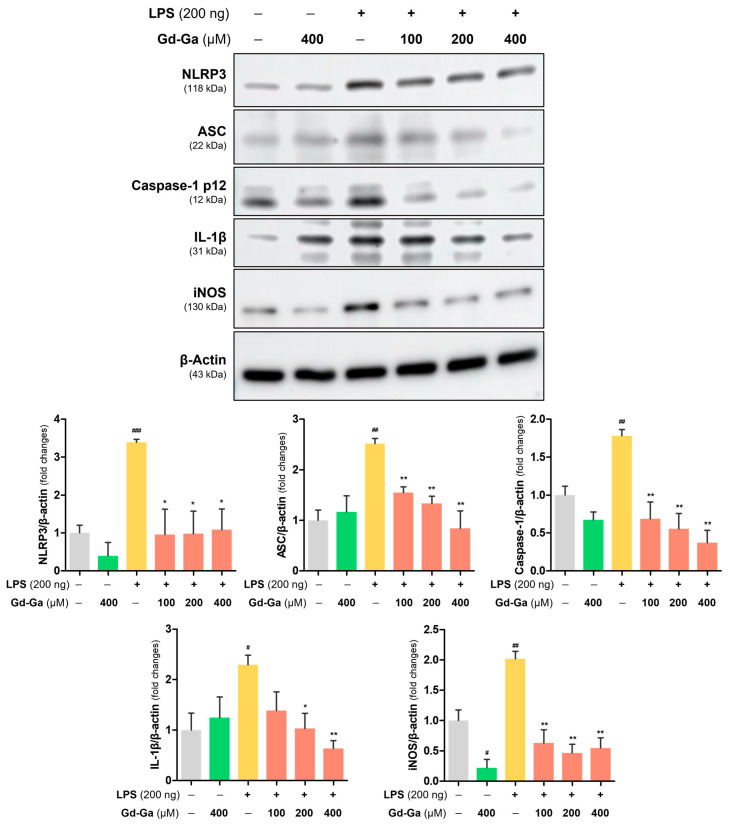
Anti-inflammatory effect in LPS-induced BV-2 cells for Gd-Ga. Representative immunoblots reacted with anti-NLRP3, ASC, caspase-1, IL-1β, and iNOS antibodies. β-actin was used as an internal loading control. Corresponding densitometric analyses of each protein’s expression levels were corrected by β-actin. Data are shown as the mean ± SEM, *n* = 3. ^#^ *p* < 0.05, ^##^ *p* < 0.01, ^###^ *p* < 0.001 compared to the control group; * *p* < 0.05 and ** *p* < 0.01 compared to the LPS-stimulated group.

BV-2 cells, stimulated by LPS, exhibited overexpression of pro-inflammatory mediators, namely NLRP3, ASC, caspase-1, and IL-1β. The quantitative data of protein expression levels were graphed compared to controls. Conversely, the group treated solely with Gd-Ga without LPS did not show a statistically significant increase in these proteins in BV-2 cells. But treatment with LPS and various concentrations (100, 200, and 400 μM) of Gd-Ga led to a decrease, indicating a significant suppression of inflammatory factors induced by LPS (Figure 6).

Furthermore, as shown in Figure 6, BV-2 cells stimulated with LPS produced an excessive amount of inducible NO synthase (iNOS), resulting in the activation of NO production and further inflammation [39,40]. Notably, in LPS-induced cells treated with Gd-Ga, the protein expression level of iNOS was markedly suppressed, as demonstrated by western blotting.

### 3.8. In Vivo MR Study on LPS-Induced Neuroinflammatory Model

**Figure 7:**
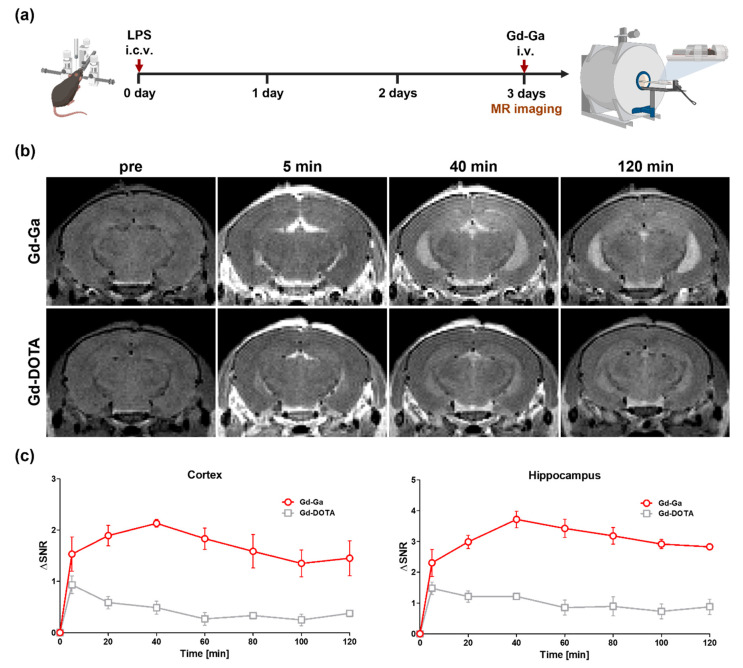
In vivo T_1_-weighted 9.4 T MR images of LPS-induced mouse models for neuroinflammation. (**a**) The schematic illustration of the experimental procedure. (**b**) Axial mouse brain images after the intravenous injection of Gd-Ga and Gd-DOTA. (**c**) The SNR differences in the cortex and hippocampus areas for T_1_-weighted images. Data are shown as the mean ± SEM, *n* = 3.

An investigation of neuroinflammation and its monitoring through MR diagnosis was conducted in 57BL/6J mice exposed to either 30 μg of LPS dissolved in 2 μL of saline (LPS group) or 2 μL of saline (control group). The administration of all Gd complexes (0.2 mmol/kg) was performed via the tail vein 72 h after LPS induction (Figure 7a). Contrast enhancements of Gd complexes at inflammatory sites were explored using a neuroinflammatory mouse model induced by LPS on a 9.4 T MRI scanner. In vivo MR studies were performed using Gd-DOTA (Figure 7b,c) and Gd-BT-DO3A (Figure S10a,b), clinically used extracellular fluid (ECF) agents, as controls. Contrast-enhanced T1-weighted MR images were obtained for 120 min after the injection of Gd complexes.

Figure 7b displayed T_1_-weighted images at 5, 40, and 120 min after the injection of Gd-Ga and Gd-DO3A. The SNR changes of Gd complexes in cortex and hippocampus regions were calculated as ΔSNR for the quantitation of contrast enhancement (Figure 7c). In the cortex, the ΔSNR value of the group treated with Gd-Ga was 2.1 at 40 min, the time of maximum contrast enhancement. The ΔSNR showed a value of 3.7 in the hippocampus at the same time. These images suggest that the contrast enhancement in the brain regions administered with Gd-Ga was higher and more persistent than that of Gd-DOTA. This pronounced contrast enhancement by Gd-Ga, distinct from Gd-DOTA in the cortex and hippocampus, strongly supported the ability for the in vivo molecular diagnosis of neuroinflammation. In the same experiment using Gd-BT-DO3A, which exhibits a higher relativity than Gd-DOTA, the ΔSNR of Gd-BT-DO3A in the cortex was 1.4 at 40 min, and in the hippocampus, it showed 1.3 at the same time. Furthermore, the contrast enhancement of Gd-BT-DO3A was not sustained and gradually decreased. These results indicated that, unlike Gd-Ga, GD-DOTA and Gd-BT-DO3A do not bind to the target protein and are therefore excreted.

Additionally, to confirm the route of drug elimination, biodistribution estimates of Gd-Ga (0.1 mmol/kg) intravenously administered into 557BL/6J mice were obtained. An ICP system was utilized to measure the Gd content in organs (heart, lung, liver, gallbladder, spleen, kidney, and intestine). Most of these were eliminated predominantly through the urine, and some via hepatic excretion (Figure S11).

### 3.9. In Vivo Anti-Inflammatory Effect of Gd-Ga on LPS-Induced Neuroinflammatory Model

**Figure 8:**
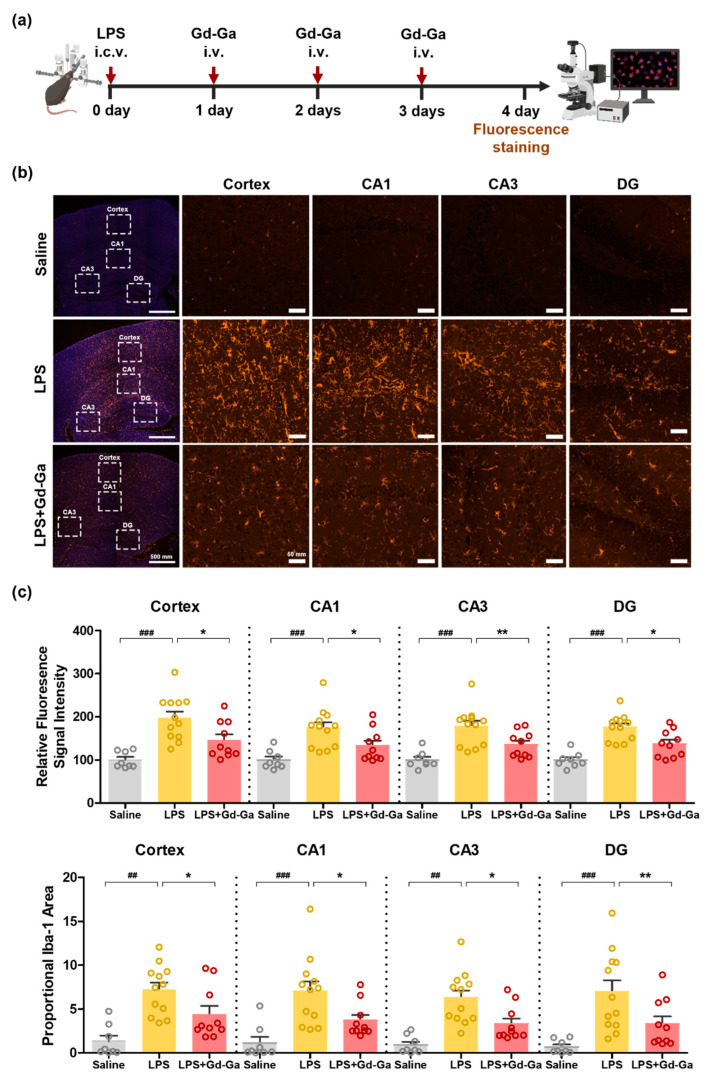
Inhibitory effect of Gd-Ga on iba-1-positive microglia in the cortex and hippocampus of LPS-induced mice. (**a**) The schematic illustration of the experimental procedure. (**b**) Immunochemistry images of mouses brain stained with iba-1 and DAPI. Higher magnification views of the boxed regions in left panels are shown. left panels; scale bar = 500 µm. Higher magnification views; scale bar = 50 µm. (**c**) The quantification graphs of data extracted from (**b**) images. Graphs obtained values from the left and right hemispheres of each mouse. Values are shown as the mean ± SEM, *n* = 8–12 from 4 to 6 mice per group. ^##^ *p* < 0.01 and ^###^ *p* < 0.001 as compared with the control group. * *p* < 0.05 and ** *p* < 0.01 as compared with the LPS group.

LPS can be employed as an intensive microglia stimulator since TLR4, a specific receptor for LPS, is localized on the surface of microglia. Inducing LPS can activate microglia, resulting in the overexpression of pro-inflammatory cytokines and astrocyte activation, ultimately leading to brain damage [1]. Iba-1 and GFAP are the most commonly used biomarkers for microglia and astrocyte activation, respectively. An increase in these factors can play a central role in various neurodegenerative disorders [41,42]. After intracerebroventricular injection of LPS (12 μg/2 μL of saline) in 557BL/6J mice, the mice were repeatedly administered Gd-Ga (0.1 mmol/kg) at 3, 24, and 48 h and sacrificed at 4 days (Figure 8a). Immunofluorescence staining of the brain section was performed with an anti-Iba-1 antibody and the anti-GFAP antibody. As shown in Figure 8b, the Gd-Ga group significantly reduced Iba-1 in the cortex, hippocampal CA1, CA3, and DG regions in LPS-induced mice. Also, the graph in Figure 8c shows Iba-1 fluorescence intensity and % area values of the Gd-Ga group diminished in the cortex, hippocampal CA1, CA3, and DG regions [43]. However, there was no change in the staining intensity of GFAP-positive astrocytes at the cortex, hippocampal CA1, CA3, and DG regions (Figure S12). This suggests that no significant differences occurred in astrocyte activation at 72 h after LPS injection.

**Figure 9:**
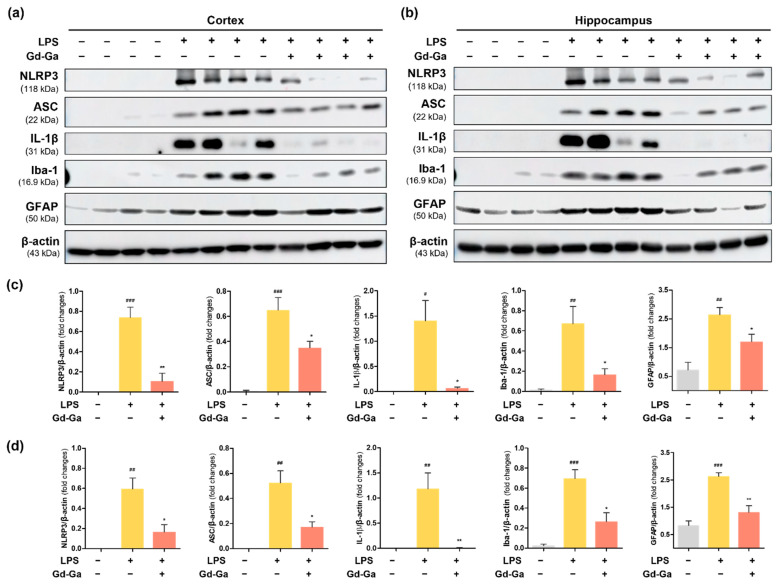
Anti-inflammatory effect of Gd-Ga in the cortex and hippocampus of LPS-induced mouse models. Western blot analysis of NLRP3, ASC, IL-1β, Iba-1, and GFAP protein levels in lysates of (**a**) cortex and (**b**) hippocampus. The graph of relative protein expression levels normalized to β-actin in (**c**) cortex and (**d**) hippocampus. Data are presented as the mean ± SEM, *n* = 4. ^#^ *p* < 0.05 compared to control group, ^##^ *p* < 0.01 compared to control group, ^###^ *p* < 0.001 compared to control group, * *p* < 0.05 compared to LPS group, ** *p* < 0.01 compared to LPS group.

Additionally, we investigated the impact of LPS stimulation on the protein expression levels of NLRP3, ASC, IL-1β, Iba-1, and GFAP in cortex and hippocampus tissues. In the LPS group treated with Gd-Ga, protein expression levels of NLRP3, ASC, IL-1β, Iba-1, and GFAP decreased in the cortex (Figure 9a,c) and hippocampus (Figure 9b,d). While LPS was effective in activating astrocytes at the protein expression level, morphological changes in astrocytes did not alter simultaneously with the observed protein expression profiles. Our findings suggest that Gd-Ga significantly attenuated LPS-induced neuroinflammation by inhibiting microglial activation and the NLRP3 inflammasome pathway.

### 3.10. Docking Study

**Figure 10:**
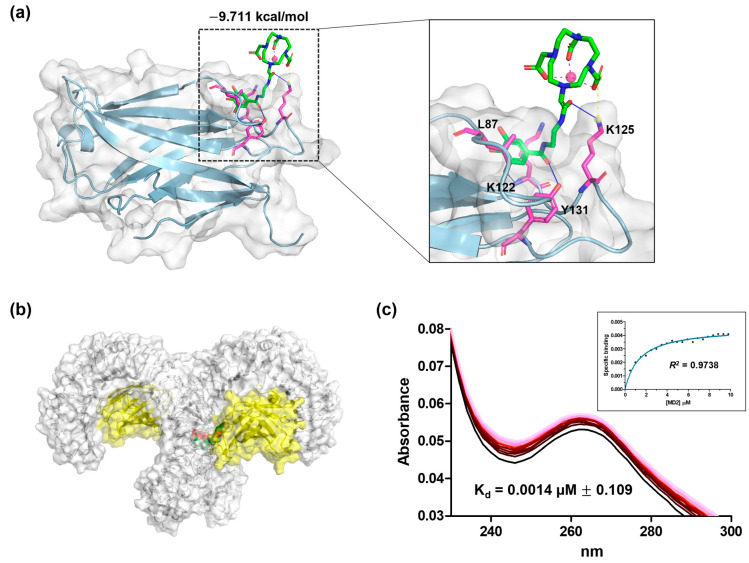
In silico study and biding affinity of Gd-Ga against MD2. (**a**) Docking pose of Gd-Ga in the binding site of MD2. (**b**) Molecular model of Gd-Ga-bound MD2 to TLR4. (**c**) The UV–vis spectra of Gd-Ga upon the addition of MD2. The inset graph represented a nonlinear curve fitting used for the calculation of K_d_.

The molecular docking was conducted to evaluate the interaction between the Gd complex and the MD2 protein, providing insights into the targeting mechanism based on in vivo results. In the pursuit of understanding the potential mechanism of Gd-Ga binding to the MD2 protein, we ran on molecular docking software to dock Gd-Ga to the crystal structure of the MD2 protein. In the presence of the MD2 surface, gallic acid conjugated with Gd chelate bound to the hydrophobic pocket, with the Gd chelate fully extending toward the external solvent, indicating a stabilized conformation [8,44]. This result can be attributed to specific interactions. The gallic acid of the Gd complex formed hydrogen bonds with Y131 and K122, which are pocket residues of MD2 and a hydrophobic interaction with L87 (Figure 8a). Additionally, the solvent-exposed portion of the Gd complex exhibited a hydrogen bond and salt bridge with K125 near the MD2 surface (Figure 8a). The binding energy (Glide Score) between Gd-Ga and MD2 was calculated to assess the advanced MD2 binding affinity. The Glide Score of Gd-Ga against MD2 was −9.711 kcal/mol. Figure 10 shows the molecular docking of Gd-Ga-bound MD2, forming a complex with TLR4. It can be assumed that it is associated with the formation of the TLR4-MD2 complex, thereby inducing an anti-inflammatory mechanism.

The direct binding affinity of Gd-Ga with MD2 was measured by a UV–vis spectrophotometer. Additions of MD2 led to changes in the intrinsic absorbance of Gd-Ga until binding saturation was reached. The equilibrium dissociation constant (K_d_) of Gd Ga was 0.0014 ± 0.109 µM (Figure 10c).

## 4. Discussion

This study aimed to validate the diagnostic and anti-inflammatory efficacy of a gadolinium complex conjugated with gallic acid for neuroinflammation (Figure 1). Gallic acid, categorized as a phenolic acid among polyphenols, features at least a single hydroxyl group on an aromatic ring structure. The most typically used classification of polyphenols divides them into two classes: flavonoids and non-flavonoid polyphenols [45]. Non-flavonoid polyphenols encompass phenolic acids, tannins, and stilbenes. Numerous phenolic acids and derivatives have proven effective in preventing and treating conditions by inhibiting oxidative stress and inflammatory mediators in various diseases, including lung injury [46,47], hepatitis [48], cancer [49], Alzheimer’s [50], and Parkinson’s disease [51]. Phenolic acids, such as gallic acid, contain a carboxylic acid group and can contribute to the stabilization of the amide bond with the amine group. In the synthesis of Gd-Ga, the carboxyl group of gallic acid was bonded to the amine group of the Gd chelate portion, thereby maintaining the antioxidant effect by preserving the hydroxyl group of the aromatic ring [52]. Excessive or prolonged production of ROS, exceeding the capacity of available antioxidant defense systems, leads to oxidative stress [53]. ROS are chemically reactive oxygen-based intermediates. ROS can react with NO, giving rise to peroxynitrite species (ONOO−), which exhibit greater toxicity than the original species [54]. Consequently, the excess ROS can either oxidize biomolecules or be involved in signaling cascades that contribute to the pathophysiology of inflammatory diseases [55]. Expanding upon this foundation, our focus zeroed in on Gd-Ga, and it markedly diminished free radicals in the DPPH and ABTS assays (Figure 3), as well as LPS-induced NO and ROS in BV-2 cells through in vitro studies, such as the Griess and DCF-DA assays (Figure 4).

Under normal conditions, when an IκB binds to an NF-κB, it sterically blocks the nuclear localization sequence, preventing the translocation of NF-κB into the nucleus. However, during inflammatory conditions induced by LPS stimulation, phosphorylation of IκBα triggers the release of NF-κB, restoring nuclear translocation [56,57,58]. Gd-Ga influenced this NF-κB signaling pathway by inhibiting the phosphorylation of IκB in the cytoplasm and blocking the translocation of NF-κB into the nucleus (Figure 5). Moreover, Gd-Ga effectively suppressed the LPS-induced expression of the NLRP3 inflammasome pathway in BV-2 cells (Figure 6) [59].

In LPS-induced neuroinflammatory mouse models, we validated that Gd-Ga reduced the protein expression levels of pro-inflammatory factors (NLRP3, ASC, and IL-1β) and glial activation (Iba-1 and GFAP) using western blotting (Figure 9). Additionally, immunofluorescence staining of Iba-1-positive cells indicated that microglia activation was suppressed in the Gd-Ga treatment group compared to the LPS-only treatment group (Figure 8). However, the staining result of activated astrocytes did not correlate with western blotting (Figure S12). Other studies have shown similar limitations. Norden et al. [60] obtained results that mRNA levels of GFAP increased 12 to 24 h after peripheral injection of LPS, but staining data showed neither differences in morphology nor immunoreactivity of GFAP. D.L. Herber et al. [61] reported a time-dependent glial reaction to LPS in APP mice compared to non-transgenic mice, having results that microglia were activated in both groups but astrocytes were only activated in APP mice. Hence, we recognize that there may be limitations in assessing the astrocyte state solely through GFAP labeling in the LPS-induced model. For more comprehensive insights, future studies will be necessary to include multiple approaches.

A publication has documented the binding activity of gallic or its derivatives to the MD2 co-receptor. Additionally, we conducted absorbance analysis using proteins to calculate the binding affinity. Experimental confirmation revealed that Gd-Ga interacts with MD2, as evidenced by changes in the absorbance of Gd-Ga with increasing MD2 concentration (Figure 10c). In the pursuit of understanding the potential mechanism of Gd-Ga binding to the MD2 protein, molecular docking simulations were conducted, wherein Gd-Ga was docked to the crystal structure of the MD2 protein. The gallic acid portion of the Gd chelate bound to the hydrophobic pocket of MD2, forming hydrogen bonds with Y131 and K122—residues within the pocket of MD2—and engaging in hydrophobic interactions with L87 (Figure 8a) [62]. The Gd chelate portion, fully extending toward the external solvent, exhibited a hydrogen bond and a salt bridge with K125 near the MD2 surface (Figure 8a). In summary, these results suggest that Gd-Ga may inactivate the TLR4/NLRP3 inflammasome pathway by binding with the MD2 protein and mitigate ROS accumulation, resulting in suppressing LPS-mediated inflammatory actions [63,64]. Further studies are needed to comprehensively understand the anti-inflammatory mechanism, along with exploring the antioxidant capacity of Gd-Ga. Nevertheless, the findings of this study imply that Gd-Ga may hold potential benefits in inhibiting the TLR4/NLRP3 inflammasome pathway.

In this study, our aim was to develop a contrast agent with anti-neuroinflammatory activity for molecular MR imaging [65]. This Gd complex enabled the detection of inflammatory lesions in LPS-induced neuroinflammatory mouse models. In Figure 7, when compared to Gd-DOTA and Gd-BT-DO3A, ECF contrast agents without specific binding, it is evident that contrast enhancement remains high. Based on previous experimental results, it can signify that, unlike the control group, Gd-Ga persists in the lesion area for an extended period due to binding to MD2.

## 5. Conclusions

This study presents the strategic design and synthesis of an MR theranostic agent, Gd chelate conjugated with gallic acid, denoted as Gd-Ga, showcasing remarkable efficacy in MRI, coupled with antioxidant and anti-inflammatory effects against neuroinflammation. Neuroinflammatory sites in LPS-induced mice were enhanced after intravenous Gd-Ga injection at the 9.4 T MR system, revealing Gd-Ga’s superior and sustained contrast enhancement in brain regions compared to Gd-DOTA and Gd-BT-DO3A. Gd-Ga exhibited potent antioxidant activity derived from rich electron donors, demonstrating remarkable free radical scavenging activities in phantom studies and effective scavenging of NO and ROS in LPS-treated BV2 cells. In vitro, Gd-Ga demonstrated the inhibition of NF-κB nuclear translocation by suppressing IκB phosphorylation, along with the attenuation of NLRP3 inflammasome activation. We further showed that Gd-Ga significantly attenuated microglial activation and inhibited the NLRP3 inflammasome pathway in the LPS-induced neuroinflammation mouse model. However, there was a limitation related to insufficient evidence for astrocyte activation in the LPS-induced mouse model, suggesting a need for future exploration in this aspect. The docking study and binding affinity results suggest that Gd-Ga is likely to modulate TLR4 expression by binding to MD2. This interaction is predicted to inhibit NLRP3 inflammasome activation, resulting in a subsequent reduction of cytokine release. This, in turn, results in the suppression of microglial activation, preventing neuronal damage. The conclusion underscores the potential application of this molecular imaging complex as an efficient platform for diagnosing and treating neuroinflammation.

## Figures and Tables

**Table 1:** The r_1_ and r_2_ relativities of Gd-Ga and Gd-DOTA in deionized water on 3.0 T and 9.4 T systems at 25 °C.

Cas	3.0 T MRI		9.4 T MRI	
	r_1_ (mM^−1^s^−1^)	r_2_ (mM^−1^s^−1^)	r_1_ (mM^−1^s^−1^)	r_2_ (mM^−1^s^−1^)
Gd-Ga	4.04 ± 0.17	4.82 ± 0.21	4.14 ± 0.11	4.78 ± 0.07
Gd-DOTA	3.40 ± 0.07	3.88 ± 0.16	3.63 ± 0.07	4.77 ± 0.11

Values are expressed as mean ± SD (*n* = 3).

**Table 2:** The EC_50_ values of antioxidant activities in the DPPH and ABTS assays for Gd-Ga, gallic acid, and ascorbic acid.

	DPPH Assay		ABTS Assay	
	EC_50_ (μM)	R^2^	EC_50_ (μM)	R^2^
Gd-Ga	4.01 ± 0.06	0.9936	8.04 ± 0.06	0.9650
Gallic acid	3.04 ± 0.13	0.9999	5.75 ± 0.01	0.9902
Ascorbic acid	9.03 ± 0.06	0.9978	17.40 ± 0.04	0.9775

Values are expressed as mean ± SEM (*n* = 3).

## Data Availability

Data are contained within the article and supplementary materials.

## Supplementary Materials

The following supporting information can be downloaded at https://www.mdpi.com/article/10.3390/antiox13020204/s1, Figure S1. ^1^H NMR spectrum of compound **2**, Figure S2. ^13^C NMR spectrum of compound **2**, Figure S3. HR-FAB-mass spectrum of compound **2**, Figure S4. HR-FAB-mass spectrum of **3**, Gd-Ga, Figure S5. HPLC spectrum of **3**, Gd-Ga, Figure S6. Free Gd ion test of Gd-Ga, Figure S7. Absorption and fluorescence spectra of Gd-Ga in water. λex = 250 nm, Figure S8. pH stability of Gd-Ga and Gd-DOTA, a commercial MR contrast agent, Figure S9. Cell viability of BV-2 cells in various concentration of Gd-Ga, Figure S10. In vivo T1-weighted 9.4 T MR images of LPS-induced mouse models injected Gd-BT-DO3A. (a) Axial mouse brain images. (b) The SNR differences in the cortex and hippocampus areas for T1-weighted images, Figure S11. Biodistribution of Gd-Ga in normal C57BL/6J mice, Figure S12. Fluorescence intensity of immunofluorescence staining for GFAP in the cortex and hippocampus of LPS-induced mouse brain, Figure S13. the source data of western blot in Figure 5, Figure S14. the source data of western blot in Figure 6, Figure S15. the source data of western blot in Figure 9.

## Abbreviations

TLR4	toll-like receptor 4
PAMPs	pathogen-associated molecular patterns
DAMPs	damage-associated molecular patterns
LPS	lipopolysaccharide
NLRP3	nucleotide binding and oligomerization domain-like receptor family pyrin domain-containing protein 3
MD2	myeloid differentiation protein 2
SET	single electron transfer
ROS	reactive oxygen species
iNOS	inducible nitric oxide synthase
NO	nitric oxide
IL-1β	interleukin-1β
IκBα	nuclear factor of kappa light polypeptide gene enhancer in B-cells inhibitor
NF-κB	nuclear transcription factor kappa B
ASC	apoptosis-associated speck-like protein containing a C-terminal caspase recruitment domain
i.c.v.	intracerebroventricular
i.v.	intravenous
MRI	magnetic resonance imaging
SE	spin echo
TE	echo time
NEX	number of excitations
FOV	field of view
TR	repetition time
SI	signal intensity
SNR	signal-to-noise ratio
K_d_	equilibrium dissociation constant
