# Supplementary material for: Gadolinium-Based Magnetic Resonance Theranostic Agent with Gallic Acid as an Anti-Neuroinflammatory and Antioxidant Agent
Source: Antioxidants (Basel). 2024 Feb 5;13(2):204. doi: 10.3390/antiox13020204 (PMC10885874; doi:10.3390/antiox13020204)
Supplement: Supplementary file 1 [file antioxidants-13-00204-s001.zip › antioxidants-2773910-supplementary.pdf]

Supplementary Information

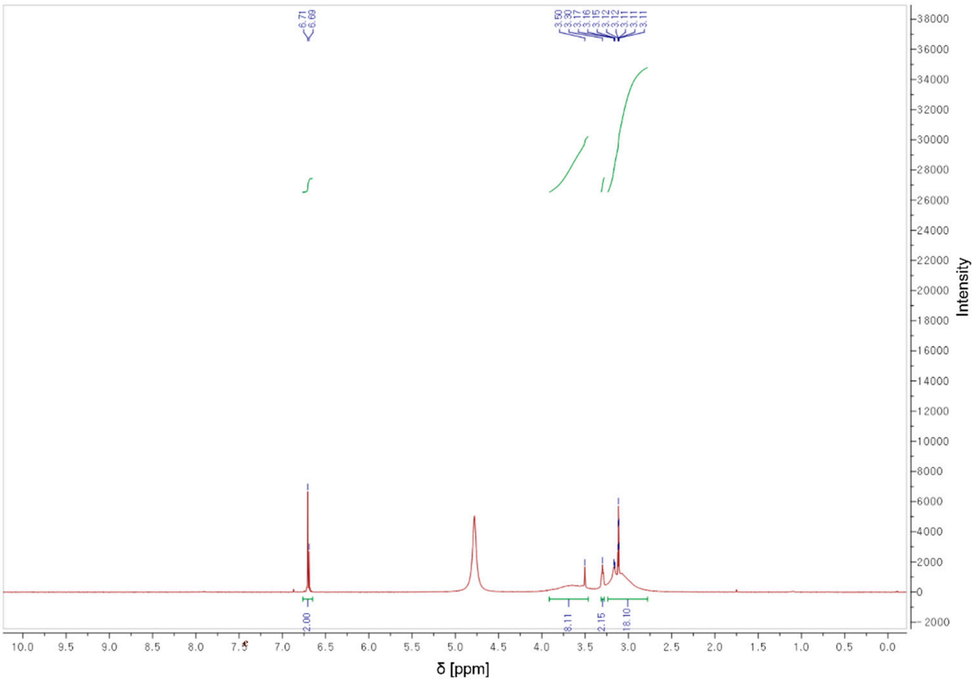

Figure S1. ^{1}H NMR spectrum of compound 2.

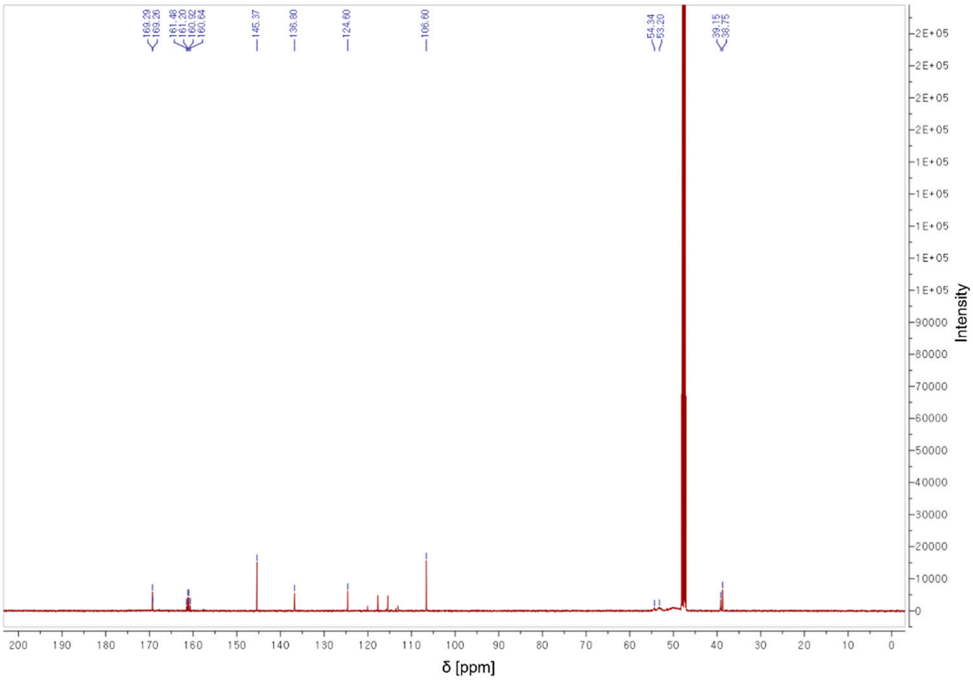

Figure S2. ^{13}C NMR spectrum of compound 2.

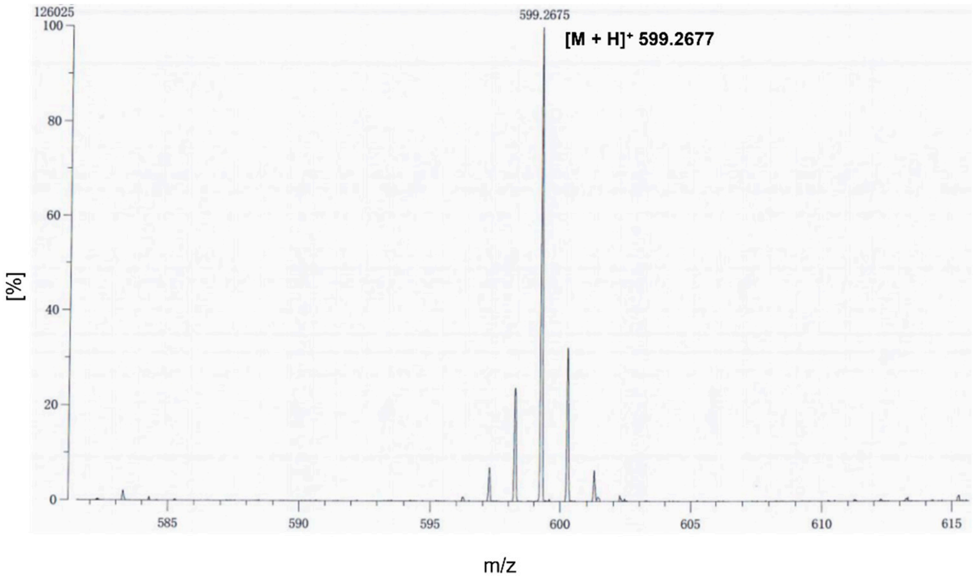

Figure S3. HR-FAB-mass spectrum of compound 2.

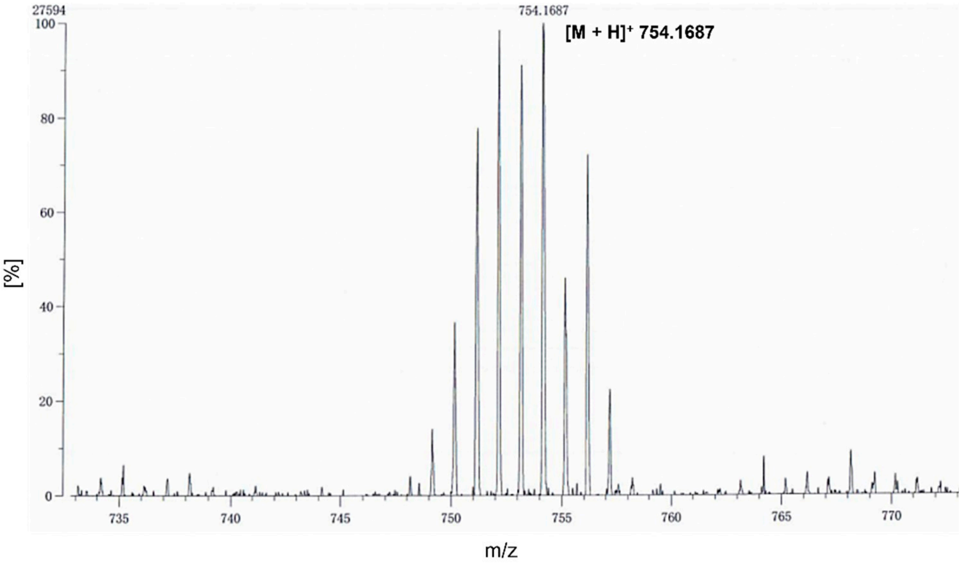

Figure S4. HR-FAB-mass spectrum of 3, Gd-Ga.

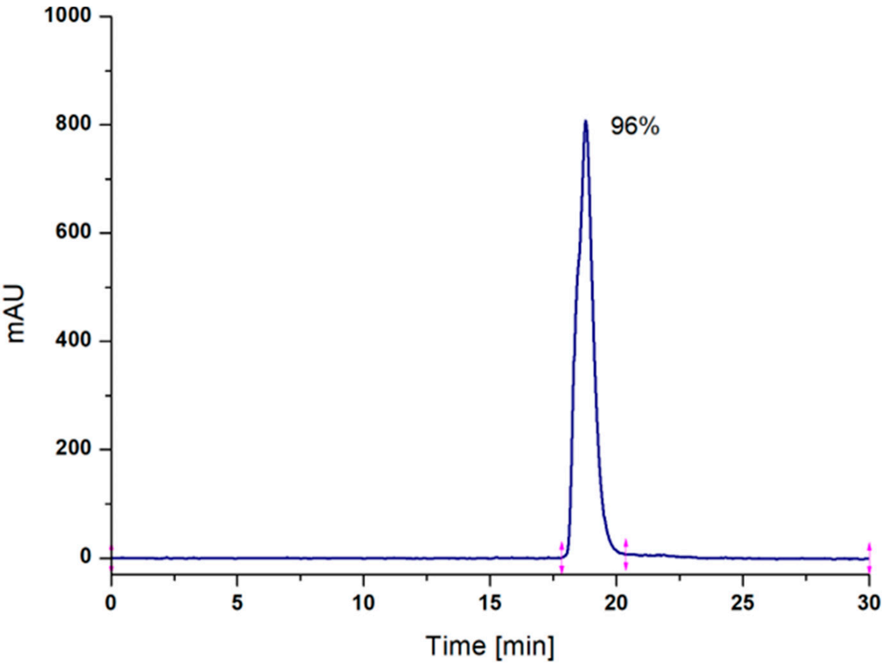

Figure S5. HPLC spectrum of 3, Gd-Ga.

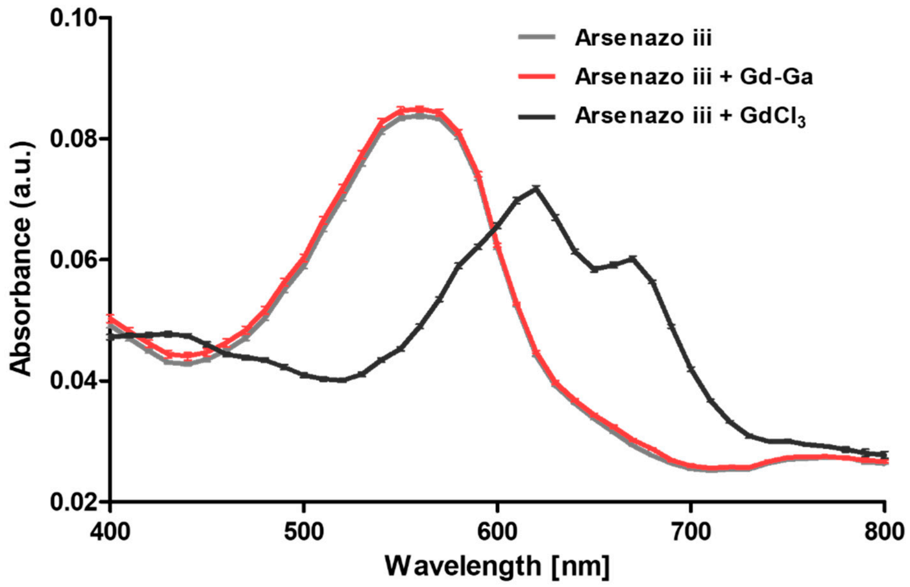

Figure S6. Free Gd ion test of Gd-Ga.

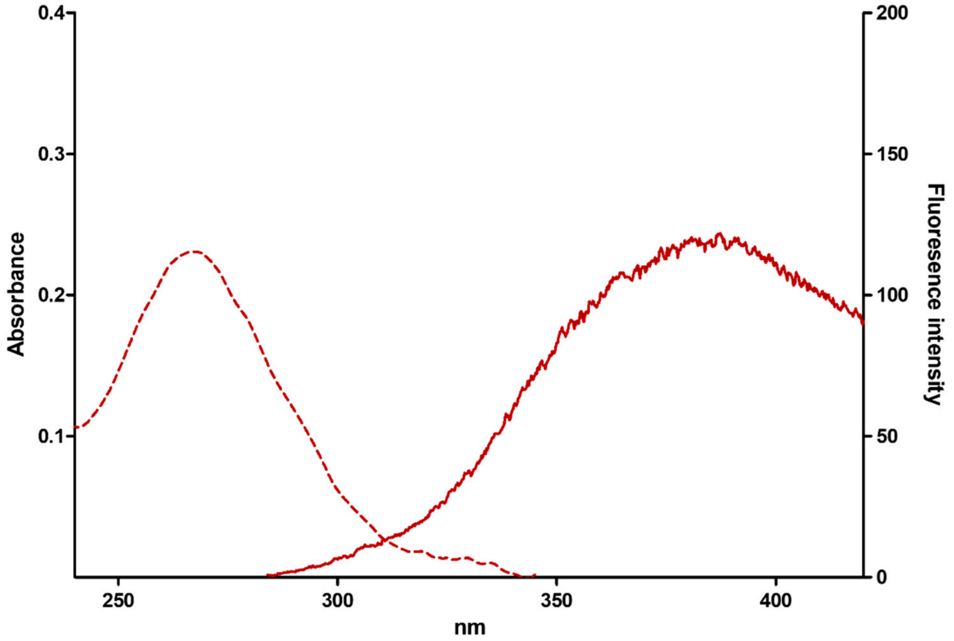

Figure S7. Absorption and fluorescence spectra of Gd-Ga in water. λ_{ex} = 250 nm.

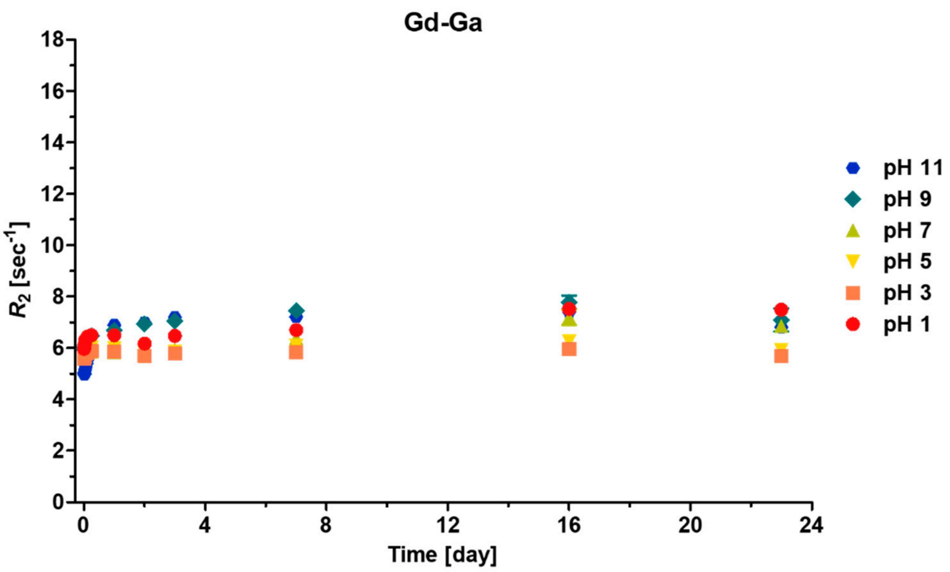

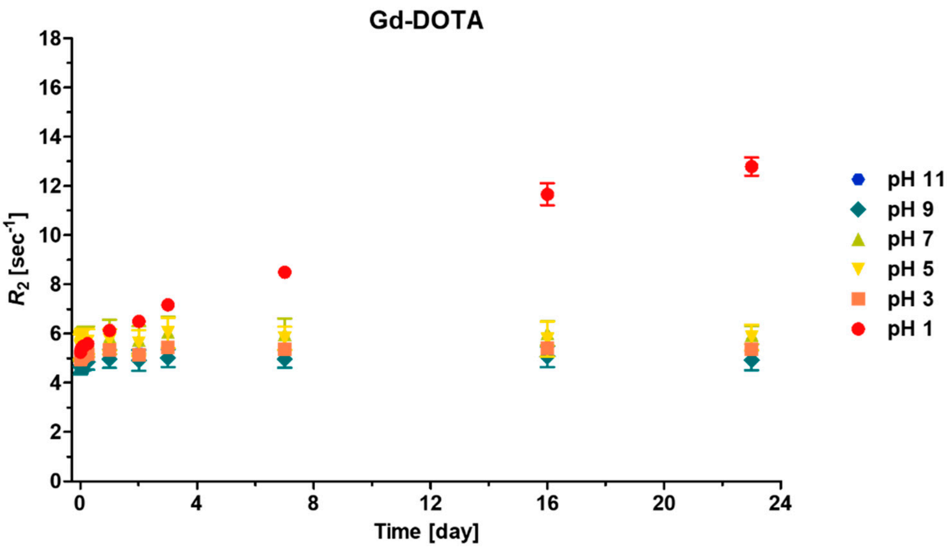

Figure S8. pH stability of Gd-Ga and Gd-DOTA, a commercial MR contrast agent.

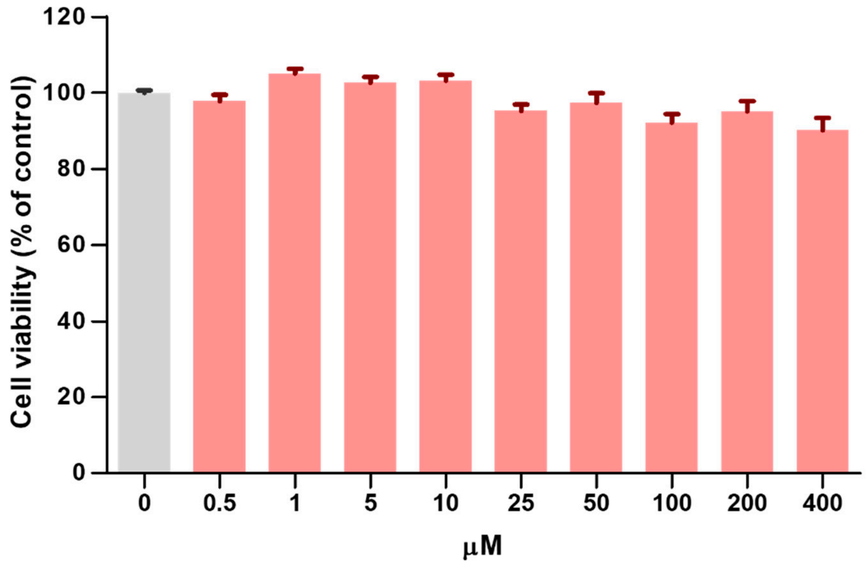

Figure S9. Cell viability of BV-2 cells in various concentration of Gd-Ga.

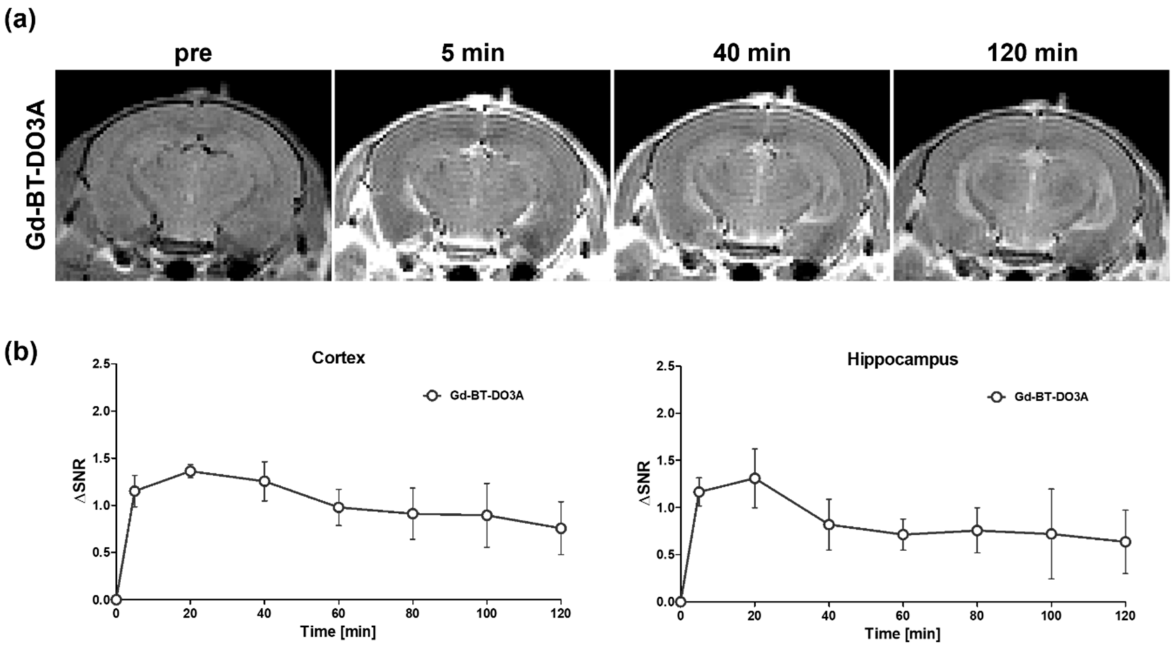

Figure S10. In vivo T_1-weighted 9.4 T MR images of LPS-induced mouse models injected Gd-BT-DO3A. (a) Axial mouse brain images. (b) The SNR differences in the cortex and hippocampus areas for T_1-weighted images.

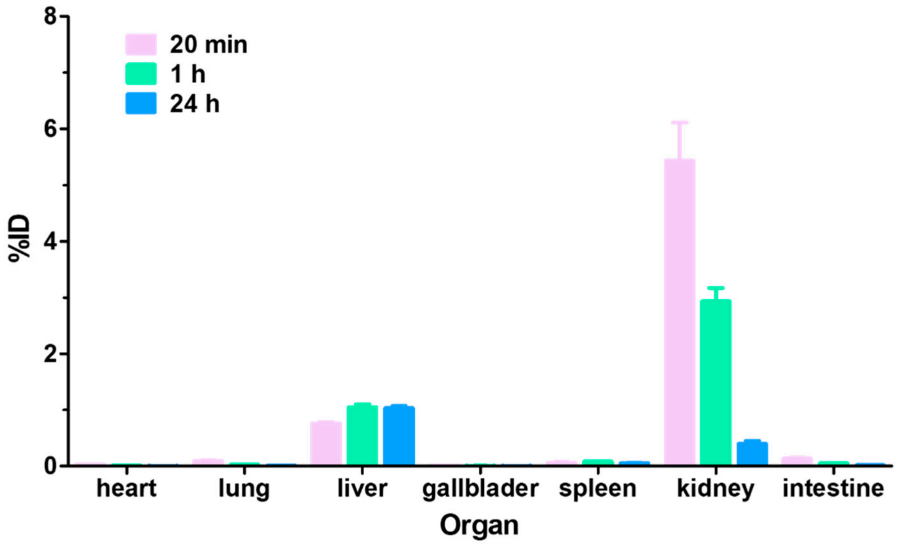

Figure S11. Biodistribution of Gd-Ga in normal C57BL/6J mice.

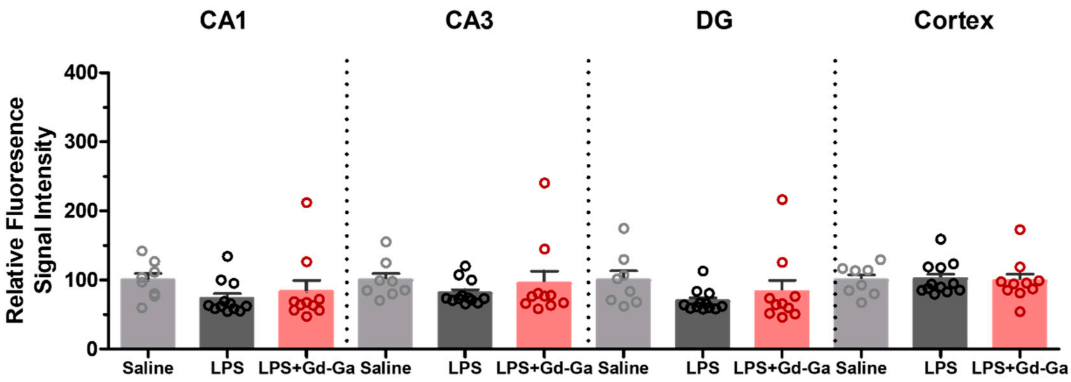

Figure S12. Fluorescence intensity of immunofluorescence staining for GFAP in the cortex and hippocampus of LPS-induced mouse brain.

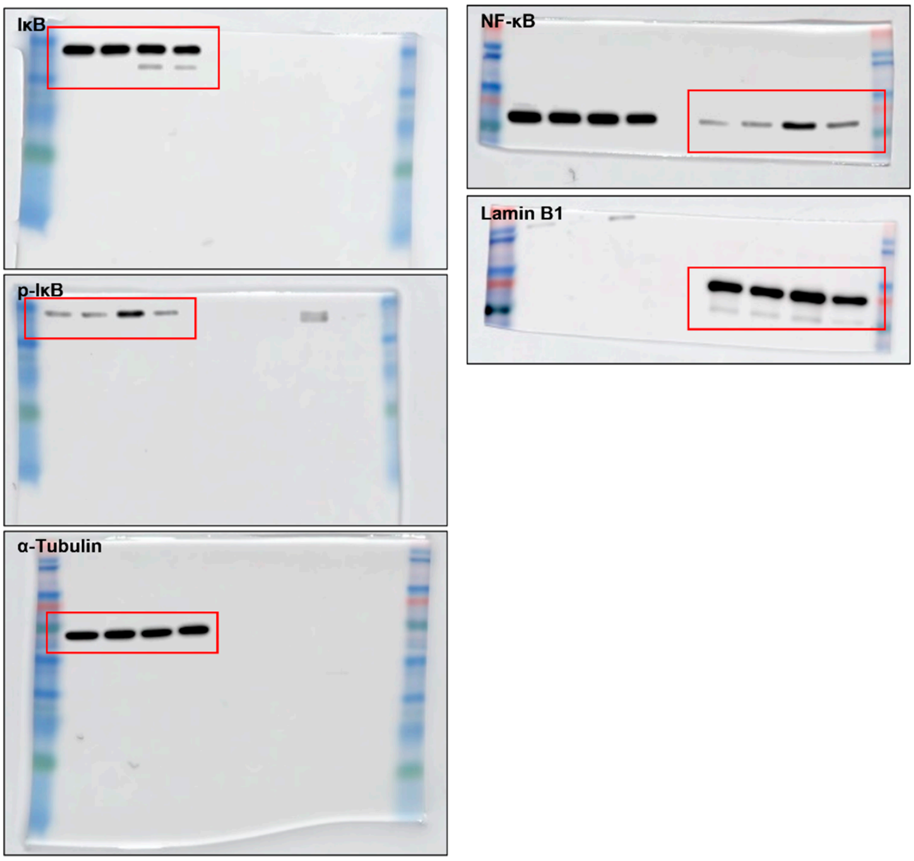

Figure S13. the source data of western blot in Figure 5.

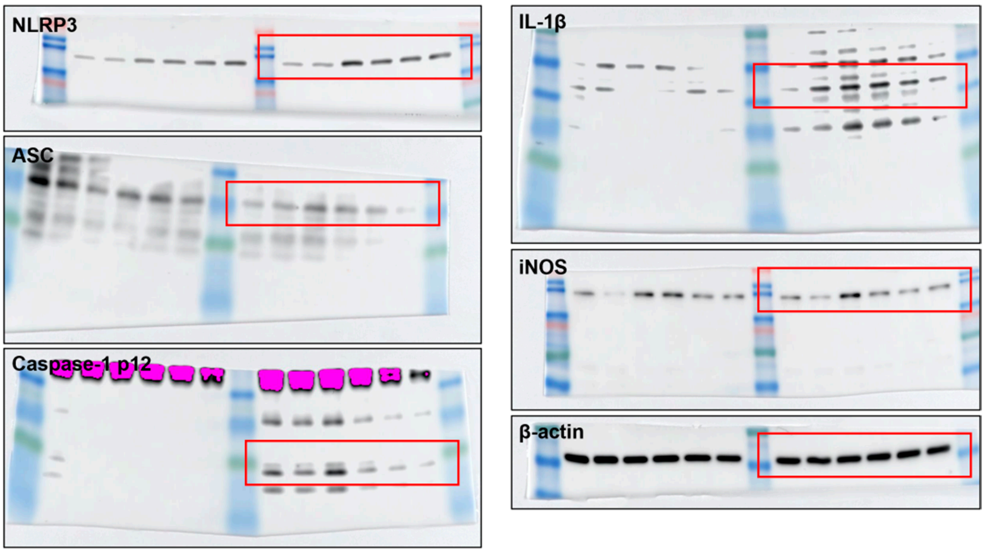

Figure S14. the source data of western blot in Figure 6.

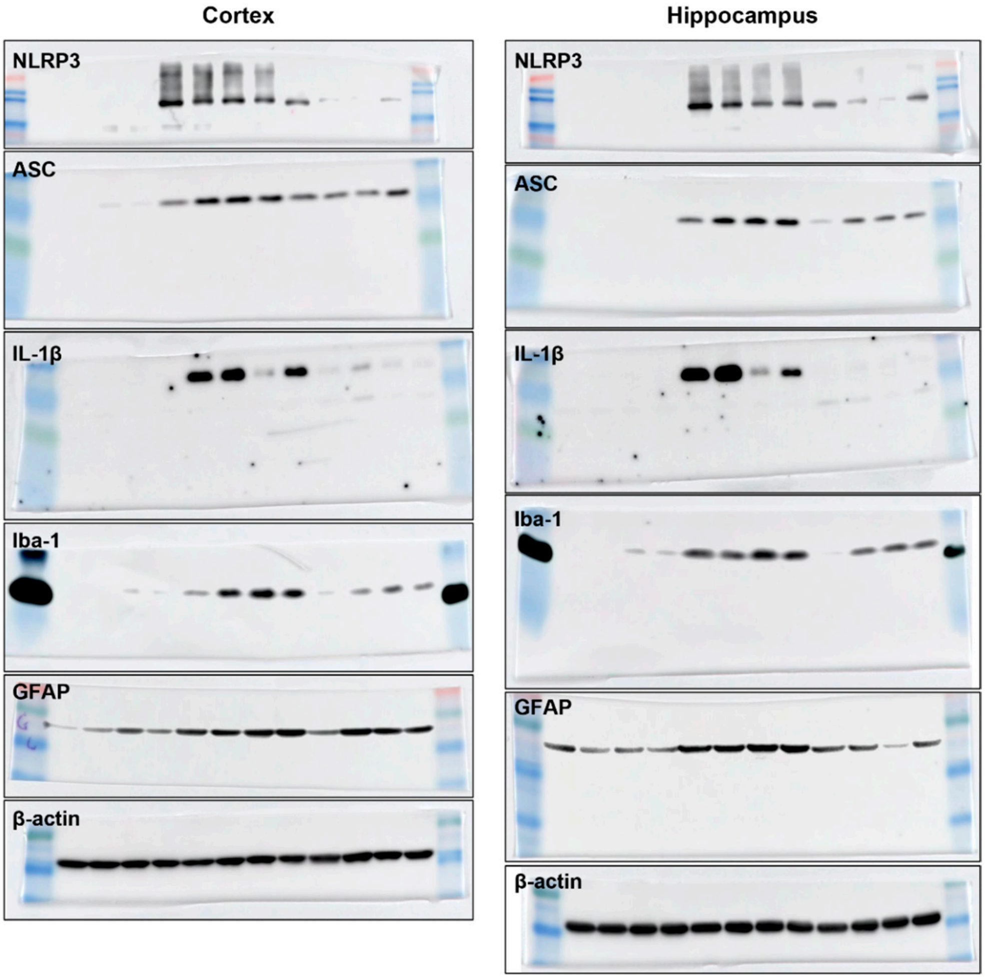

Figure S15. the source data of western blot in Figure 9.
